# Paired-End Sequencing of Long-Range DNA Fragments for *De Novo* Assembly of Large, Complex Mammalian Genomes by Direct Intra-Molecule Ligation

**DOI:** 10.1371/journal.pone.0046211

**Published:** 2012-09-27

**Authors:** Asan, Chunyu Geng, Yan Chen, Kui Wu, Qingle Cai, Yu Wang, Yongshan Lang, Hongzhi Cao, Huangming Yang, Jian Wang, Xiuqing Zhang

**Affiliations:** BGI-Shenzhen, Shenzhen, Guangdong, China; University of Oxford, United Kingdom

## Abstract

**Background:**

The relatively short read lengths from next generation sequencing (NGS) technologies still pose a challenge for *de novo* assembly of complex mammal genomes. One important solution is to use paired-end (PE) sequence information experimentally obtained from long-range DNA fragments (>1 kb). Here, we characterize and extend a long-range PE library construction method based on direct intra-molecule ligation (or molecular linker-free circularization) for NGS.

**Results:**

We found that the method performs stably for PE sequencing of 2- to 5- kb DNA fragments, and can be extended to 10–20 kb (and even in extremes, up to ∼35 kb). We also characterized the impact of low quality input DNA on the method, and develop a whole-genome amplification (WGA) based protocol using limited input DNA (<1 µg). Using this PE dataset, we accurately assembled the YanHuang (YH) genome, the first sequenced Asian genome, into a scaffold N50 size of >2 Mb, which is over100-times greater than the initial size produced with only small insert PE reads(17 kb). In addition, we mapped two 7- to 8- kb insertions in the YH genome using the larger insert sizes of the long-range PE data.

**Conclusions:**

In conclusion, we demonstrate here the effectiveness of this long-range PE sequencing method and its use for the *de novo* assembly of a large, complex genome using NGS short reads.

## Introduction

The availability of genome sequence information can greatly aid and underpin the biological research of a given species. However, even a decade after the completion of the human genome project [1]–[2], a high quality reference genome sequences remains unavailable for most mammalian species (http://www.ncbi.nlm.nih.gov/genomes/static/gpstat.html) [3]. This is mainly due to the prohibitive cost required for *de novo* sequencing and assembly of large, complex genomes using traditional Sanger sequencing. Fortunately, the recent advent of next generation DNA sequencing (NGS) [4]–[7], which produces ultra-high throughput short reads at several order of magnitude reduced unit data cost and unprecedented speed, makes whole genome shotgun sequencing (WGSS) of genomes more cost-effective and increasingly practical. However, efforts to *de novo* assembling NGS short reads, especially for mammalian genomes that include complex repeat sequences, have been greatly limited by the read-length [8]–[11].

**Figure 1:**
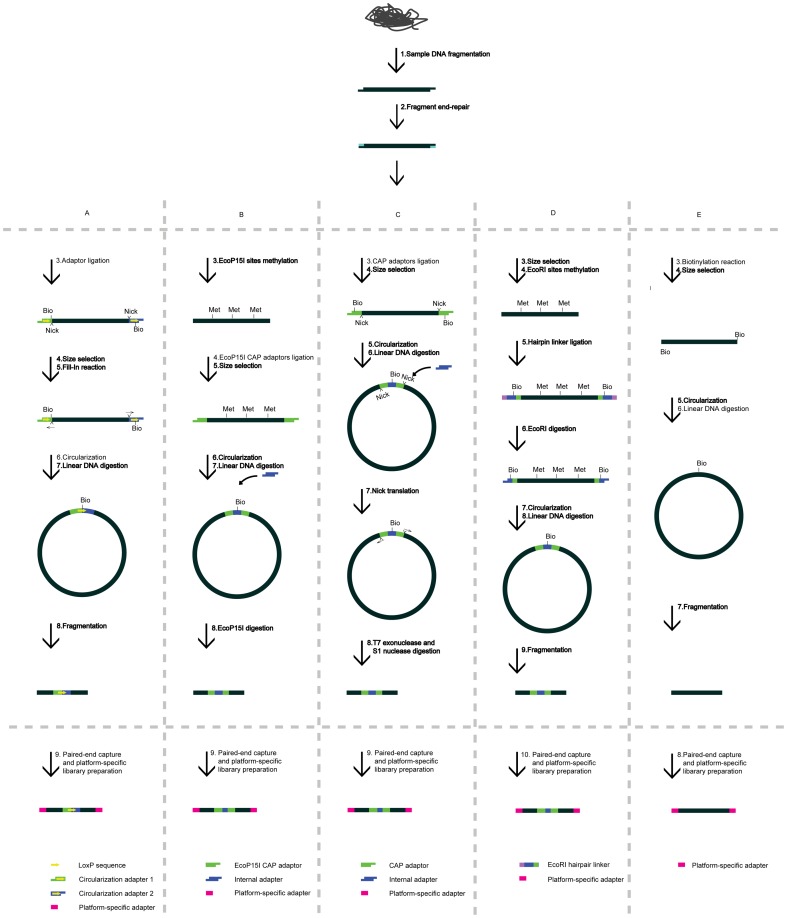
Comparison of long-range PE sequencing methods. (A–D) Long-range PE sequencing with linker oligonucleotides. In these methods, biotin-labeled linker oligonucleotides are added to the two ends of long-range DNA fragments, followed by enzymes-induced intra-molecule circularization, and recovery of the paired-end for sequencing. The addition of linker oligonucleotides and subsequent complex enzyme reactions require 5–8 recoveries before capturing the paired-ends from circularized DNA fragments. In addition, the use of expensive enzymes involves additional costs. (E), Long-range PE sequencing by direct intra-molecule ligation or molecular linker-free circularization. In the method, the 3′ends of long-range DNA fragments were biotin-labeled, followed by direct intra-molecule circularization and recovery of PE ends. This method requires less recovery steps (3–4) and no complex enzyme reaction system. The steps for DNA recovery are in bold. We applied the method E in this research.

One potential solution to this issue is to perform hierarchical assembly using paired-end (PE) sequence from different classes of long-range DNA fragments. For short DNA fragments, typically 200–800 bp in size, PE sequences can be easily achieved by direct sequencing from two ends of standard short insert libraries [6]. However, for the long-range DNA fragments required by the assembly strategy, the two ends of a DNA fragment must be joined together to sequence the junction fragments. Several methods have been developed to achieve this purpose [12]–[15], where biotin-labeled linker oligonucleotides are added to the two ends of a targeted DNA fragment to covalently join (or circularize) them using enzyme-induced ligation (Fig. 1 A–D). Though generally effective, the scalable applications of these methods are limited by two weaknesses (Table S1). First, the introduction of linker oligonucleotides and subsequent complex enzyme reactions require additional experimental steps that reduce the efficiency of mass conversion into sequencing-compatible materials, and as a result of losses during these steps, more input DNA is required [15]. Moreover, this loss becomes increasingly serious for longer fragments. Second, the necessary addition of expensive enzymes, such as Cre recombinase and *Eco*P15I, exert additional costs for the library preparation. A potential alternative method is linker-free by directly circularizing blunt-ended DNA fragments by intra-molecule ligation using common DNA ligase (Fig. 1 E and Table S1) [6]. Recently, we have successfully used this method for the *de novo* sequencing and assembly of a typically complex mammalian genome [16],i.e. the giant panda genome. However, the performance of the technique has not been well-characterized to date.

To further explore the efficiency of our technique for PE sequencing of long-range DNA fragments, here we characterized and extended the method. We assessed the performance of this method for PE sequencing of ≤5 kb DNA fragments by triplicate experiments, and also explored its utility with longer-range DNA fragments (10, 20 and 35 kb). To characterize the starting material issues, we tested the impact of low quality input DNA on the method and developed a whole-genome amplification-based (WGA-based) protocol for using limited input DNA (<1 µg). Finally, we demonstrated the power of this PE sequencing method for *de novo* genome assembly by accurately improving the assembly of the YanHuang (YH) genome, which is the first sequenced Asian genome [17], from a scaffold N50 size of an initial 17 kb (from using only the short insert PE reads) to 2 Mb (by integrating the long-range PE data). Furthermore, we also attempted to identify examples of long-insertions in the YH genome by taking advantage of the larger insert sizes of the PE data. These results represent a significant step forward in the application of this method to related studies.

## Results

### The method and key performance parameters for consideration

The key experimental steps in the long-range PE sequencing method are illustrated in Fig. 1 E. Briefly, to determine PE sequences separated by long distances, DNA fragments of the required length were blunt-ended, biotin-labeled at both 3′ ends and circularized by direct intra-molecule ligation. These steps were followed by removal of the remaining linear fragments, fragmentation of the circularized DNA and separation of the biotin-labeled junction fragments, and downstream platform-specific library preparation for PE sequencing (steps nearly identical to the standard small insert library preparation). In accord with these experimental steps, several parameters were considered for the performance evaluation. These performance parameters included: the contamination of small insert reads (∼500 bp) originating either from un-digested linear DNA fragments or from fragments with damaged sites (nicks) within circularized molecules that were labeled by biotin-dNTPs; the introduction of chimerically ligated DNA fragments during DNA circularization; the introduction of PCR duplicates due to the reduced library complexity, and the library complexity that could be measured by the final number of PE molecules with distinct origins that have proper insert sizes and orientation relationships when mapped to the human genome.

### Performance for ≤5 kb sized fragments

**Figure 2:**
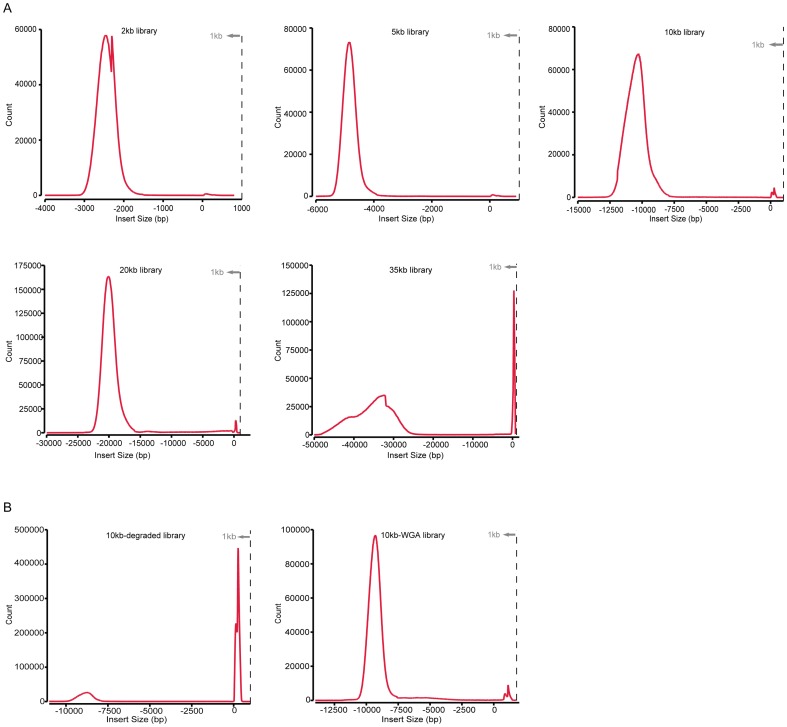
Insert-size distributions of long-range PE sequencing libraries. (A), 2- to 35-kb libraries; (B), 10 kb-WGA and 10 kb-dam libraries. The read-pairs that were uniquely mapped to the human genome (NCBI build 37) were used for this analysis. The insert size of a library and its corresponding small insert read contamination are shown in the ‘−’ and ‘+’direction of the x-axis, respectively. The ‘−’ direction represents the orientation relationship between PEs from circularized long-range DNA molecules (>1 kb) when mapped to the human genome, while ‘+’ represents that between the two ends from linear small DNA fragments (∼500 bp).

Considering the practicalities and the necessities of protocol optimization, we initially tested our method for constructing PE libraries using ≤5 kb DNA fragments. We were able to obtain a stable yield of libraries using the protocol outlined in the *Materials and Methods*, and successfully constructed ∼2 kb and ∼5 kb spanning libraries with 3 replicates for each. For each library, we generated 7–10 million PE reads of high quality data on Illumina sequencing platforms (see the *≤5 kb* section in Table 1, and methods outlined in the *Material and Methods*), which presents a sufficient physical coverage of at least 9-fold over the human genome for the performance analyses. To determine the baseline level of the performance parameters for the standard small insert PE library, we also included the data of two PE libraries of ∼500 bp in insert size from YH project [17] in the comparison (see the *500 bp* section in Table 1). For these two standard small insert libraries, we observed PCR duplicate rates of <0.2%, mapped read pairs rates of ∼90% and uniquely mapped read pairs rates of <85%, and of the uniquely mapped read pairs a >98% rate of properly mapped reads (accounting for ∼83% of the total reads) and a <1.4% rate of chimerical reads. In contrast, for the two types of long-range PE libraries, we found a <4.3% rate of PCR duplicates, a >47% rate of mapped read pairs and a >37% rate of uniquely mapped read pairs, and of which >95%, <1% and <4.5% was properly mapped read pairs (accounting for 35–50% of the total reads), small insert reads contamination and chimerical reads, respectively (Table 1, the section of *<5 kb*). Thus, compared to the standard small insert PE library, although there was a general reduction in the performance due to its complex preparation process, the long-range PE sequencing method can robustly produce highly complex libraries for ≤5 kb DNA fragments. As a further support of this observation, we also found both types of long-range PE libraries showed insert sizes of highly consistency with the desired lengths (2-kb libraries: 2.3 kb in mean size with standard variation <260 bp; ∼5-kb libraries: 4.8–5.4 kb in mean size with standard variation <280 bp) (Table 1, the section of *≤5 kb*; and Fig. 2 A). Furthermore, given the nature that the impact of the level of small insert reads contamination and chimerical reads could be ruled out using supporting information among read pairs in specific applications, these results demonstrate the efficiency of the PE sequencing method for ≤5 kb DNA fragments. However, in inter-comparison, we found the 5 kb libraries showed a better performance than the 2 kb libraries (Table 1, the section of *≤5 kb*).

**Table 1:** The performance of long-range PE sequencing libraries.

	Insert size (SD; bp)	Number of reads (M)	Duplicate rate (%)	Mapped read pairs (%)	Uniquely mapped read pairs (%)	Percent of uniquely mapped read pairs*		
						Proper (%)	Small (%)	Chimeric (%)
*500 bp*								
500 bp-1	482 (13)	21.81	0.17	90.49	84.84	99.03	—	0.97
500 bp-2	520 (16)	22.74	0.15	88.62	82.87	98.61	—	1.39
*≤5 kb*								
2 kb-1	2301 (233)	9.54	2.77	47.22	36.79	95.26	0.56	4.18
2 kb-2	2300 (209)	7.5	3.08	47.62	37.46	95.08	0.65	4.27
2 kb-3	2302 (256)	7.68	2.56	51.56	39.51	97.21	0.29	2.50
5 kb-1	4855 (242)	7.36	4.05	54.26	51.55	96.53	0.35	3.12
5 kb-2	4854 (252)	7.14	4.32	51.01	47.1	94.45	1.01	4.54
5 kb-3	5475 (275)	8.03	3.93	51.16	48.1	95.45	0.40	4.15
*10–35 kb*								
10 kb	10,278(788)	20.02	9.27	61.87	57.41	95.58	0.70	3.72
20 kb	19,979(1120)	27.05	51.82	36.03	32.31	92.19	0.64	7.17
35 kb	33,358(5235)	16.31	53.46	33.67	23.82	47.84	6.45	45.71
*Testing input gDNA*								
10 kb-dam	8,816(470)	20.4	14.31	68.98	54.14	23.27	72.99	3.75
10 kb-WGA	9,579(521)	24.12	29.34	56.04	49.54	80.53	1.63	17.84

* Proper, properly mapped long-range PE reads; Small, small insert PE reads contamination; and Chimeric, chimerical reads.

Duplicates were defined as PE reads that have precisely the same sequences on both ends; the duplication rate was calculated by one minus the percent of the non-redundant PE reads in total reads. For each library, the normal long-range PE reads were defined as the uniquely mapped read pairs that have correct orientation relationships and insert-sizes lying within ±3SD; small insert read contamination represent the uniquely mapped read pairs that have the orientation relationships and insert sizes of a standard ∼500-bp spanning library; and chimeric reads represent the remaining reads excluding the above two classes of read-pair.

### Performance for ≥10 kb sized fragments

To further test our method for constructing longer-range PE libraries, we developed protocols for circularizing ∼10-, ∼20- and ∼35-kb DNA fragments (see in *the Materials and Methods*). We subjected one PE library for each of the ∼10-, ∼20- and ∼35-kb fragments to HiSeq2000 sequencing (PE 45) to produce 16–27 million high quality reads each (see the *10–35 kb* section in Table 1), which represent a physical coverage of at least 80-fold over the human genome. In comparison with the 2 kb and 5 kb libraries, we found that the 10-, 20- and 35-kb library displayed PCR duplicate rates of ∼9.27, ∼51.82 and ∼53.46% (compared to the rate of <4.3% of the 2 kb and 5 kb libraries), mapped read pairs rates of ∼62%, ∼36% and ∼34% (compared to the rate of >47% of the 2 kb and 5 kb libraries), and uniquely mapped read pairs rates of ∼57%, ∼32% and ∼34% (compared to the rate of >37% of the 2 kb and 5 kb libraries), respectively (see the*<5 kb* and*10–35 kb* sections in Table 1). Of the uniquely mapped read pairs, we observed for the 10-, 20- and 35-kb library, properly mapped read pairs rates of ∼96% (accounting for ∼55% of the total reads), ∼92% (accounting for∼30% of the total reads) and ∼49% (accounting for ∼12.4% for the total reads) (compared to the rate of >95%, accounting for >35% of the total reads, of the 2 kb and 5 kb libraries), small insert reads contamination rates of 0.7%, 0.7% and 6.5% (compared to the rate of <0.7% of the 2 kb and 5 kb libraries), and chimerical reads rates of ∼3.7%, ∼7.0% and ∼45.7% (compared to the rate of >2.5% of the 2 kb and 5 kb libraries), respectively. Thus, there was a further systematic reduction of performance of the method (including increased PCR duplicate rate, reduced number of properly mapped read pairs (or library-complexity), and increased rate of small reads contamination and chimerical reads) for ≥20 kb DNA fragments. However, we found that the results are even acceptable for the 35-kb library based on several lines of facts: first, the insert sizes of the three libraries were highly consistent with experimental expectations (∼10-kb library: 10,278 bp in mean size with standard variation <790 bp; ∼20-kb library: 19,979 bp in mean size with standard variation <1,200 bp; ∼35-kb library: 33,358 bp in size with standard variation <5,300 bp) (see the <5 kb and 10–35 kb sections in Table 1, and Fig. 2 A); second, the levels of small insert size reads contamination and chimeric reads can be ruled out for specific applications (these read pairs are always orphans without supporting information from others); the substantial reductions of properly mapped read pairs showed very limited impact on the randomness or uniformity of sequence coverage over the human genome (Figure S1 and S2). Taken together, these results indicated that though the performance further systematically reduced with longer DNA fragments, the method was even effective for 35 kb DNA fragments.

Note that in inter-comparison, we found the 5-kb and 10-kb libraries showed a better performance than other libraries (Table 1, the sections of *≤5 kb* and *10–35 kb*), as may be related with our more concentrated efforts in experimental protocol optimization for these two types of libraries.

### The impact of input DNA

The quality and quantity of DNA samples were two main prerequisites for constructing long-range PE sequencing libraries. Longer insert sizes always require higher quality and quantity of the input DNA. To address these two important issues, we also prepared ∼10-kb libraries for mildly damage-treated DNA (10 kb-dam) and DNA from WGA of 500 ng starting material (10 kb-WGA). By comparing the performance with the normal 10-kb library, we sought to observe the intrinsic impacts of the two treatments on the method (see the *10–35 kb* and *testing input DNA* sections in Table 1).

For the 10 kb-dam library, we found a 5% increased PCR duplicates rate (14% compared to 9%), a 3% decreased rate of uniquely mapped read pairs (54% compared to 57%), and of the uniquely mapped read pairs, a comparable level of chimerical read pairs (3.7% compared to 3.7%) and a dramatically increased rate of potential small reads contamination (73% compared to 0.7%) and leaving a dramatically decreased rate of properly mapped reads (23% compared to 96%) which accounts for only 13% of the total reads (compared to 55% of the normal library) (see the sections of *10–35 kb* and *Testing input DNA* in Table 1, and Fig. 2). Thus, low quality DNA could induce a dramatic increase of the small reads contamination (73% of the uniquely mapped read pairs, presumably arising from the mis-labeling of biotinylated dNTPs at nicks within the DNA fragments during library preparation) and correspondingly a substantial decrease of the library-complexity (13% of the total reads) for the long-range PE sequencing method, therefore may poses a serous challenge for a specific application.

In contrast, for the 10 kb-WGA library, we found a greater increase level of PCR duplicates rate (20%: 29% compared to 9%), a higher decrease level of the uniquely mapped read pairs (8%: 49% compared to 57%), and of the uniquely mapped read pairs, a greater increase level of chimerical reads (14%: 18% compared to 4%) but still a ∼80% rate of properly mapped reads accounting for 40% of the total reads (compared to 55% of the normal library) (see the sections of *10–35 kb* and *Testing input DNA* in Table 1, and Fig. 2). Thus, for WGA-based library preparation, although there was a substantial increase of chimerical reads (18% of uniquely mapped read pairs, presumably induced during the WGA process), the method still display a high level of library-complexity (40% of the total reads). We further looked at the chimerical reads and found that 88.7% of the chimeric reads (15.8% out of 18%) in WGA library were from intra-chromosome abnormal ligation while the rest were from inter-chromosome. However, for a specific application, there is a necessary for eliminating the impact of the chemical reads.

Taken together, these results indicate that the quality of input DNA is of key importance for the method, and WGA that is a good compromise for a limited input DNA source after eliminating the effect of the effects of chimerical reads.

### De *novo* assembly of the YH genome

**Figure 3:**
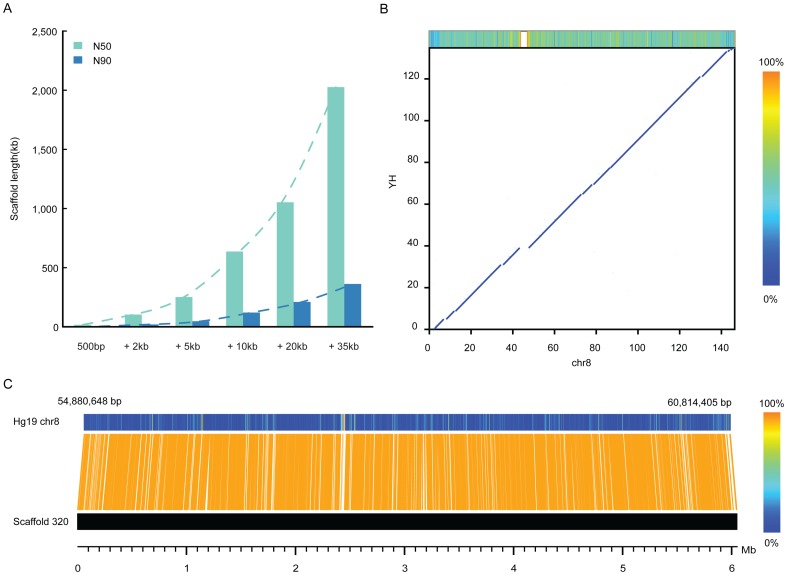
*De novo* assembly of the YH genome. (A), The YH scaffold N50 (green bar) and N90 (blue bar) sizes were dramatically improvement with the addition of long-range PE information (from 2 kb to 35 kb). The trends of improvement are shown as a dashed line. (B), Alignment between the assembled YH scaffolds (y-axis) and the reference human genome (NCBI build 37, x-xis) on chr8. Local repeat level in the reference chr8 (calculated in a 1-kb window) is showed in color along the chromosome at the top-up bar. The white blocks in the bar represent the gaps in the reference genome. (C), Alignment of the YH scaffold 320 onto the reference chr8. Local repeat level on the region of the reference chr8 is also shown in color along the sequence (calculated in a 1-kb window).

To assess the method's power for *de novo* assembly, we used the five classes of long-range PE reads (2, 5, 10, 20 and 35 kb, but not the 10 kb-dam and 10 kb-WGA) to improve YH genome assembly. Starting from an initial contig N50 size of ∼1 kb and scaffold N50 size of ∼17 kb with 52-fold short-insert PE reads (200∼500 bp) from the YH project and following studies [8], [17], we added the long-range PE information (from 2 kb to 35 kb) step by step using the SOAPdenovo assembler [8]. At each stage, we considered two contigs in a scaffold only if there were more than 5 read-pairs supporting their connection. We found that the scaffold size displayed a dramatic improvement with the addition of long-range PE information: from a N50 size of 17 kb with <500 bp PE reads to 2.1 Mb with 35 kb PE reads, and a corresponding N90 size from 3.5 kb to 360 kb (see Table 2 and Fig. 3 A). Corresponding to this improvement in scaffold size, we also found a remarkable improvement in the final contig size (N50 from 1 kb to 8.5 kb, and N90 from 200 bp to 1.7 kb), a 8% improvement in the final genome coverage (from 80 to 88%) and a 2% improvement in the final gene coverage (from 95 to 97%). To assess the accuracy of assembly, we next aligned the assembled scaffolds against the reference human genome (NCBI build 37) (see *Materials and Methods*). In general, the YH scaffolds matched the reference human genome with good continuity and coverage (Fig. 3 B and C), except for some gaps at highly repetitive genome regions such as centromeres. There were only 115 of the 37,000 scaffolds (∼0.3%) aligned to different regions on the reference genome. Because structural variants in the YH genome also induce abnormal alignments between the two genomes, we assumed the rate of incorrectly assembled scaffolds was <0.3%, and most of which could be corrected by mapping the related PE reads back to these scaffolds. Taken together, these results indicate the potential power and high reliability of integrating PE reads in the de novo assembly of mammalian genomes.

**Table 2:** Summary of *de novo* YH genome assembly.

	Sequence depth (fold)	Physical coverage (fold)	N50 length (bp)	N90 length (bp)	Total length (Gp)	Scaffold (contig) Number	Genome Coverage (%)	Gene Coverage (%)
Contig	52.1	-	1,050	205	2.15	3884.491	80.72	95.80
Scaffold (+<500 bp PE)	31.6	115	17,061	3,534	2.54	335,245	80.72	95.80
Scaffold(+2 kb PE)	7.7	192	103,604	20,240	2.70	92,913	80.72	95.80
Scaffold(+5 kb PE)	6.2	380	251,707	47,121	2.81	56,411	80.72	95.80
Scaffold(+10 kb PE)	4.2	748	636,400	120,228	2.89	42,591	80.72	95.80
Scaffold(+20 kb PE)	0.8	181	1,052,921	210,357	2.91	39,059	80.72	95.80
Scaffold (+35 kb PE)	0.2	88	2,026,143	362,377	2.93	37,028	80.72	95.80
Contig after gap closure	-	-	8,536	1,669	2.55	853,305	88.01	97.43

The data from the YH project was used for the contig and initial scaffold assembly. Then, the long-range PE data were added step by step for scaffold construction. Genome coverage and gene coverage was calculated using the NCBI build 37 and RefSeq gene set as reference, respectively. The X and Y chromosomes were excluded while calculating genome coverage and gene coverage. For calculation of scaffold N50, N90 and total length, the intra-scaffold gaps were included.

### Long insertions detection for the YH genome

**Figure 4:**
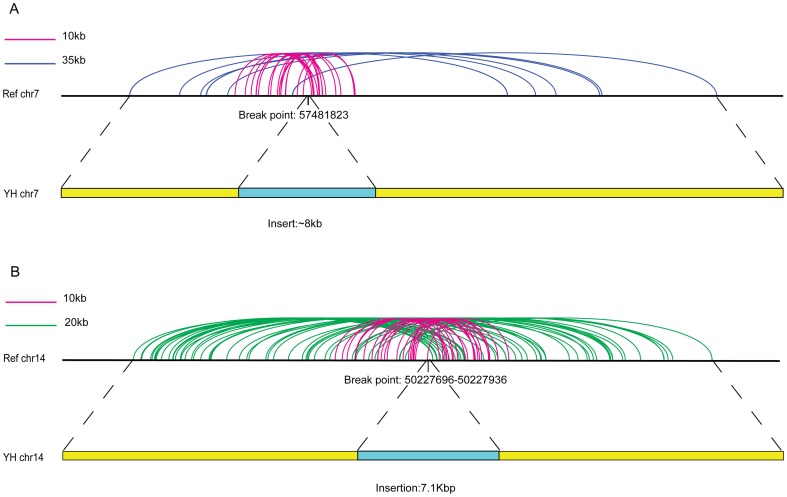
Two long insertions in YH genome detected by long-range PE. Mapping the long-range PE reads back to the human genome (NCBI build 37) resulted in the detection of a previously identified ∼8 kb insertion in chromosome 7 (A) and a novel ∼7 kb insertion in chromosome 14 (B) in the YH genome. The abnormally mapped PE reads that supported the insertions by showing unexpected short insert size are shown.

Utilizing the advantage of larger insert-sizes, long-range PE reads (>1 kb) provide a complementary way to use the short insert PE reads (∼500 bp) for mapping longer insertions in genomes. To date, the structural variants (SVs) of the YH genome, including long insertions, have been extensively characterized [17]–[19], but there is no sophisticated bioinformatics pipeline available to detect SVs using long-range PE data. Thus, to demonstrate this potential application of long-range PE sequencing, we mapped our PE reads (not including the10 kb-dam and 10 kb-WGA data) back to the human reference genome and looked for clusters of PE reads with exceptionally short insert sizes that could support long insertions in the YH genome. We show two examples of these results in Fig. 4 (7–8 kb): in the first example, a known ∼8 kb insertion in Chr7 was confirmed with a cluster of exceptionally short insert-sized PE reads from 10-kb and 35-kb libraries (Fig. 4 A); in the other example, a novel ∼7 kb insertion in chromosome 14 was detected with a similar cluster of exceptionally short insert-sized PE reads from 10-kb and 20-kb libraries (Fig. 4 B), the size of which was subsequently validated by following PCR study (Fig. S4). Thus, with more sophisticated bioinformatics pipelines in the future, using long-range PE reads in combination with standard short PE reads could be an effective way to map long insertions in human genomes.

## Discussion

*De novo* sequencing and the assembly of large, mammalian genomes with short NGS reads generally requires PE sequence information from long-range DNA fragments. Thus, methods for preparing long-range PE sequencing libraries for ultra-high-throughput sequencing play an important role in an assembly strategy. In this study, we evaluated an experimental method that is linker-free and methodologically based on direct intra-molecule ligation (molecular-linker free circularization) using inexpensive ligases. This unique feature confers the method the advantage of a higher efficiency of mass conversion of input DNA into sequencing-compatible materials and/or a lower cost over alternatives.

The insert size is one of the most important factors that affect the efficiency of long-range PE library construction using this method. Despite the general efficiency of the method, we observed a systematic reduction in performance with an increase of insert size (the trend was especially seem for ≥10 kb libraries), including an increased rate of small reads contamination, chimeric reads and duplicated reads, as well as decreased library complexity. This is highly consistent with the fact that longer DNA molecules are more difficult to recover and circularize, and correspondingly the final library complexity is reduced. In spite of the impact of longer insert sizes, we were still able to prepare PE sequencing libraries for up to 35 kb DNA fragments with acceptable complexity using this method. This insert-size is nearly comparable to those of fosmid-cloning, and is the longest ever reported by such a method. Considering the larger amount of input DNA required, the labor and time consumption, low success rate and a higher cost of the fosmid-cloning [20]–[21], our method could be used as an alternative for related studies in the future.

The starting material is another important factor for consideration in long-range PE library construction. Compared to small PE library preparation, long-range PE sequencing generally required a higher quality and quantity of starting materials. This is the reason that the preparation of long-range PE libraries involves many molecular manipulations leading to certain proportions of the DNA samples not being recovered at each step, meaning that only a very high quality (i.e., high molecular-weight) and high quantity of starting materials can guarantee a sufficient yield for the final library. We demonstrated for the method that, due to the unique features of biotin labeling, input DNA quality is of exceptional importance: low quality input DNA can substantially increase the proportion of small insert reads contamination (presumably derived from mislabeling at nicks in circularized DNA fragments). We also developed a WGA-based protocol for preparing high quality PE sequencing libraries using limited starting material (<1 µg), which is important for related studies with limited amounts of DNA.

The ultimate goal of this study was to develop an effective PE sequencing method to improve *de novo* assembly of large, complex genomes using NGS. Using an established hierarchical assembly pipeline [8], [16] and YH genome assembly as an example, we showed that the addition of long-range PE reads information can lead to a 100-times improvement of the scaffold N50 sizes (from 17 kb to 2.0 Mb), and a final improvement of ∼7% and ∼2% for genome and gene coverage, respectively. This level of scaffold size is even comparable with that size in the initial stage of the human genome project [1]. In addition, we showed using a criterion of at least 5 PE reads supporting a connection in assembly that 99.7% of the YH scaffolds could be correctly mapped back to the reference genome. Furthermore, PE mapping the reads to the reference human genome enabled us to identify long insertions (>1 kb), exemplified by a known 8-kb insertion in chr7 and an novel 7-kb insertion in chr14 of the YH genome. These sizes of insertions can be characterized at very high sequencing depth using only short insert PE reads by assembly.

In addition, our method has very promising features and potentially a much wider scope and utility in other types of sequencing studies. First, given the simplicity of experimental steps and its performance, the method can be readily adapted to other NGS platforms, such as 454 and SOLID, simply by just adding the platform-specific adaptors in the step after capturing the junction fragments of the circularized molecules. Second, in addition to the application in de novo assembly and SV mapping and given the nature of long-range PE sequencing, the method could potentially be adapted to other research contexts, such as discovering transcript structure, identifying regulatory and epigenetic elements, and revealing chromatin interactions.

In summary, we characterized and extended a versatile long-range PE sequencing method for NGS. Our results demonstrated the effectiveness of the long-rang PE sequencing method and its use in *de novo* assembly of large, complex genomes with NGS short reads.

## Materials and Methods

### Genomic DNA preparation

High molecular weight (HMW) genomic DNA (100–200 kb in size) was extracted from a YH [17] lymphoblastoid cell line using proteinase K and phenol chloroform [22]. The molecular weight and quantity of DNA were examined using pulsed field gel electrophoresis analyses (PFGE; Bio-Rad, Hercules CA) and Quant-iT dsDNA HS Assay kit quantification (0.2–100 ng) (Invitrogen, Q32854, Carlsbad CA), respectively. For 10 kb-WGA library construction, sufficient quantity of DNA (>30 µg) was derived from an initial aliquot of 500 ng genomic DNA by whole genome amplification using a REPLI-g Mini Kit (Qiagen, 150025, CA) following the manufacturer's instructions. For 10 kb-dam library construction, 30 µg HMW genomic DNA was repeatedly treated at mildly degrading conditions, i.e. frozen at −80°C for 20 min and thawed at 70°C for 20 min 5 times.

### Long-range PE libraries preparation

#### Input DNA and fragmentation

Input DNA quantities of 20, 20, 30, 50 and 50 µg were used for 2-, 5-, 10-, 20- and 35-kb PE libraries construction, respectively. Genomic DNA (10 µg) was fragmented using a HydroShear DNA shearing device (GeneMachines, San Carlos CA) in 150 µl TE buffer (Qiagen) with following settings (for 2 kb: standard-assemble/speed-code 9/25 cycles; for 5 kb: large-assemble/speed-code 6/25 cycles; for 10 kb: large-assemble/speed-code 10/20 cycles; for 20 kb: large-assemble/speed-code 13/30 cycles; and for 35 kb: large-assemble/speed-code 15/40 cycles). The fragmented DNA was cleaned up using a QIAquick PCR purification kit (Qiagen, 28106) (for fragments ≤10 kb) or AMPure SPRI beads (Agencourt, Beverly MA) (for fragments ≥20 kb).

#### End-repair and Biotin-labeling

Purified DNA fragments were end-repaired with a dNTP mixture using an enzyme mixture including T4 DNA polymerase (Enzymatics, Beverly MA), T4 PNK (Enzymatics), and Klenow DNA Polymerase (Enzymatics). After a second round of clean-up (methods seen in the above step for *after fragmentation purification*), the 3′ ends of the DNA fragments were labeled with a dNTP mixture including 15% biotin labeled nucleotide analogues (PerkinElmer Inc., Boston MA) using the same enzymes as used for end-repair.

#### Size selection

DNA fragments for targeted sizes were subsequently selected after agarose gel electrophoresis (Certified Megabase Agarose; Bio-Rad): for 2-kb library, with 0.8% gel at 85 V for 4 h; for 5-kb library, with 0.6% gel at 35 V for 16 h; for 10-kb library, with 0.5% gel at 35 V for 16 h; for 20-kb library, with 0.4% gel at 27 V for 18 h; and for 35-kb library, with 1% gel by PFGE (3.5 V/cm, switch time 0.1–10 s, and run-time 24 h). The size-selected DNA fragments were recovered from gel slices using a QIAquick PCR gel extraction kit (Qiagen, 28706) (≤10 kb) or QIAEX II gel extraction kit (Qiagen, 20051) (≥20 kb).

#### Circularization, linear-DNA digestion and fragmentation

Approximately 600–1000 ng targeted DNA fragments were subjected to circulation for each library construction by direct intra-molecule ligation using either T4 DNA-ligase (Enzymatics) or T3/T4 DNA-ligase (Enzymatics) (incubating at 16°C for 16–18 h). The targeted DNA concentrations of the circularization reaction were different: for 2 kb/5 kb, 2 ng/µl; for 10 kb, 1 ng/µl; for 20 kb, 0.5 ng/µl; for 35 kb, 0.25 ng/µl. The remaining linear DNA fragments were removed by Exonuclease I (NEBiolabs, Ipswich MA) and Plasmid-safe ATP-dependent DNase (Epicentre, Madison WI). After denaturing the enzymes (at 75°C for 15 min), the circular DNA was fragmented to approximately 500 bp in size by sonication using an adaptive focused acoustic machine (Covaris, Woburn MA) with the following parameters: for 2–10 kb, frequency sweeping/intensity 10/10% duty cycle/1000 bursts per second/4.5 min; for the 20 kb and 35 kb library, frequency sweeping/intensity 10/10% duty cycle/1000 bursts per second/6 min.

#### Post-fragmentation library construction

Biotinylated DNA fragments were purified using M280 streptavidin beads (Invitrogen). After end-repair, A-tailing and Illumina PE adapters ligation (following the Illumina standard protocol for small insert-size library preparation), the DNA was amplified from beads using Phusion Polymerase (NEB) with PCR conditions: 40 sec at 98°C, 18 cycles of (10 sec at 98°C, 30 sec at 65°C, 40 sec at 72°C), and 5 min at 72°C. Library fragments of 450–650 bp in length were recovered from 2% agarose gels followed by validation using an Agilent 2100 Bioanalyzer (DNA 1000 Kit; Agilent, Santa Clara CA) and qPCR quantification for sequencing (The sequence data has been deposited in the NCBI Short Read Archive with accession number SRA057128).

### Sequencing, data process and short read mapping

Each library was bar-coded and sequenced on a Genome Analyzer II*x* or HiSeq2000 as paired-end 45- or 90-bp reads (PE45 or PE90). Base-calling was performed using the Illumina pipeline (version1.3.4). Reads of lower quality (having >60% bases of a low Phred quality (<8) or .>5 ‘N’ nucleotides) or adaptor contaminations (having >10 bases aligned to the adapter sequences allowing 3 bp mismatches) were filtered together with the paired reads. The read pair duplicates (read pairs that precisely duplicated others) were removed before mapping (only one pair was retained). The remaining read pairs were mapped onto the reference human genome (NCBI build 37) using the SOAP aligner [23]–[24] allowing up to 1 mismatch at either end.

### Definition of performance parameters in data statistics

PCR duplicates, read pairs that have precisely the same starting mapping positions of both ends to the human genome; the mapped read pairs; read pairs with both ends mapped to one locus or multiple loci on the human genome; the uniquely mapped read pairs, read pairs mapped to only one locus on the human genome; the properly mapped read pairs, the uniquely mapped read pairs that could be mapped to the human genome with correct orientation-relations and insert sizes lying within mean ±3SD; the small read contaminations, the uniquely mapped read pairs that could be mapped to the human genome with orientation-relationships and insert sizes expected by a standard small PE library; the chimeric read-pairs, the uniquely mapped read pairs that could be mapped to the human genome with abnormal orientation-relationships(mapped to different chromosomes or different loci of one chromosome) or insert sizes lying out mean ±3SD.

### *De novo* assembly of the YH genome and whole genome alignments

The YH genome was assembled using an established pipeline utilizing the SOAPdenovo assembler [8]. An initial set of contigs was constructed using 52-fold short insert size (<500 bp) data from the YH project [17] and following work [8] with the de Bruijn graph construction. The contigs were then joined into scaffolds by the step by step addition of the PE information, from 180 bp, to 500 bp, and to 2 kb, 5 kb, 10 kb, 20 kb and 35 kb; and finally gaps within the scaffolds were filled by local assembly via retrieving read pairs that had one read well-aligned to the contigs and the other read located in the gap region.

Whole-genome alignment between YH scaffolds and the reference human genome was performed using lastz [25], with the parameters: K = 2200, L = 6000, Y = 3400, E = 30, H = 0, O = 400, and T = 1. Then the Chain/Net package [25] was used for post treatment. The YH genome was masked with RepeatMasker repeats (http://www.RepeatMasker.org) against the Repbase transposable element library (version 2011-04-19) [26] and TRF tandem repeats [27] of period ≤12. The reference human genome, together with repeats annotation, was downloaded from the UCSC database (http://genomics.ucsc.edu), which has the same sequence as the NCBI build 37.

To determine the large-scale accuracy of YH assembly, we located each scaffold sequence onto the reference according to the above alignment results. If a scaffold was anchored on a chromosome, 90% of its gap-free region (excluding “N” and repeat sequences) should be aligned on the chromosome. For scaffolds mapped to different chromosomes, we mapped the related PE reads back to the scaffolds to check whether these scaffolds were chimeric resulting from the assembled method or if there were real chromosome rearrangements in the YH genome.

## Supporting Information

Figure S1 Sequence-depth coverage on chromosome 8 of standard small insert PE reads (blue dots) and long-range PE reads (red dots). Sequence depth was calculated in 10 kb-window for a total of ∼7-fold combined sequencing data from long-range PE libraries (except the 10 kb-dam and 10 kb-WGA data) and 7-fold sequencing data from standard small-insert libraries.
(DOCX)

Figure S2 Physical coverage distribution of long-range PE libraries data on human genome. A subset data of properly mapped unique read pairs that represent ∼20-fold physical coverage on the human genome for each 2-kb (red), 5-kb (blue), 10-kb (green), 20-kb (black) and 35-kb libraries (orange) was sampled (base on the total data of 35-kb library) the analysis.Poisson (no bias) distributions (gray) with λ = 20 is also shown. Poisson distribution is the expected if there were absolutely no bias.
(DOCX)

Figure S3 Alignments between the YH scaffolds and the reference human genome (Chr1–7, 9–22, X and Y).
(DOC)

Figure S4 PCR experimental validation of a newly identified ∼7-kb insertion in the YH genome chr14.
(DOCX)

Table S1 Comparison of protocols for long-range PE sequencing methods.
(XLS)

Table S2 PCR primer information of the insertion on chr14.
(XLS)
