# Supplementary material for: Paired-End Sequencing of Long-Range DNA Fragments for De Novo Assembly of Large, Complex Mammalian Genomes by Direct Intra-Molecule Ligation
Source: PLoS One. 2012 Sep 27;7(9):e46211. doi: 10.1371/journal.pone.0046211 (PMC3459883; doi:10.1371/journal.pone.0046211)
Supplement: Figure S1 — Sequence-depth coverage on chromosome 8 of standard small insert PE reads (blue dots) and long-range PE reads (red dots). Sequence depth was calculated in 10 kb-window for a total of ∼7-fold combined sequencing data from long-range PE libraries (except the 10 kb-dam and 10 kb-WGA data) and 7-fold sequencing data from standard small-insert libraries. (DOCX) [file pone.0046211.s001.docx]

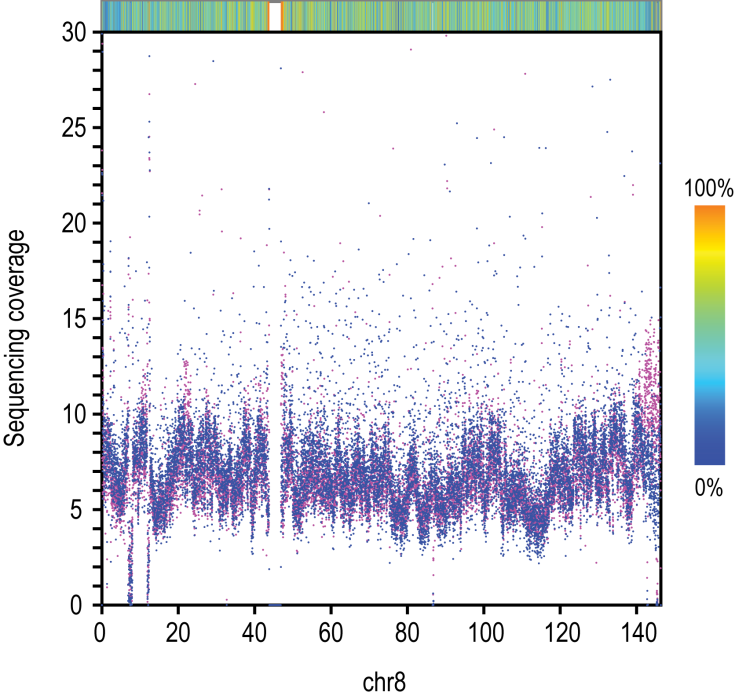


**Figure S1 Sequence-depth coverage on chromosome 8 of standard small insert PE reads (blue dots) and long-range PE reads (red dots).**Sequence depth was calculated in 10 kb-window for a total of ~7-fold combined sequencing data from long-range PE libraries (except the 10 kb-dam and 10 kb-WGA data) and ~7-fold sequencing data from standard small-insert libraries.
