# Supplementary material for: Paired-End Sequencing of Long-Range DNA Fragments for De Novo Assembly of Large, Complex Mammalian Genomes by Direct Intra-Molecule Ligation
Source: PLoS One. 2012 Sep 27;7(9):e46211. doi: 10.1371/journal.pone.0046211 (PMC3459883; doi:10.1371/journal.pone.0046211)
Supplement: Figure S2 — Physical coverage distribution of long-range PE libraries data on human genome. A subset data of properly mapped unique read pairs that represent ∼20-fold physical coverage on the human genome for each 2-kb (red), 5-kb (blue), 10-kb (green), 20-kb (black) and 35-kb libraries (orange) was sampled (base on the total data of 35-kb library) the analysis.Poisson (no bias) distributions (gray) with λ = 20 is also shown. Poisson distribution is the expected if there were absolutely no bias. (DOCX) [file pone.0046211.s002.docx]

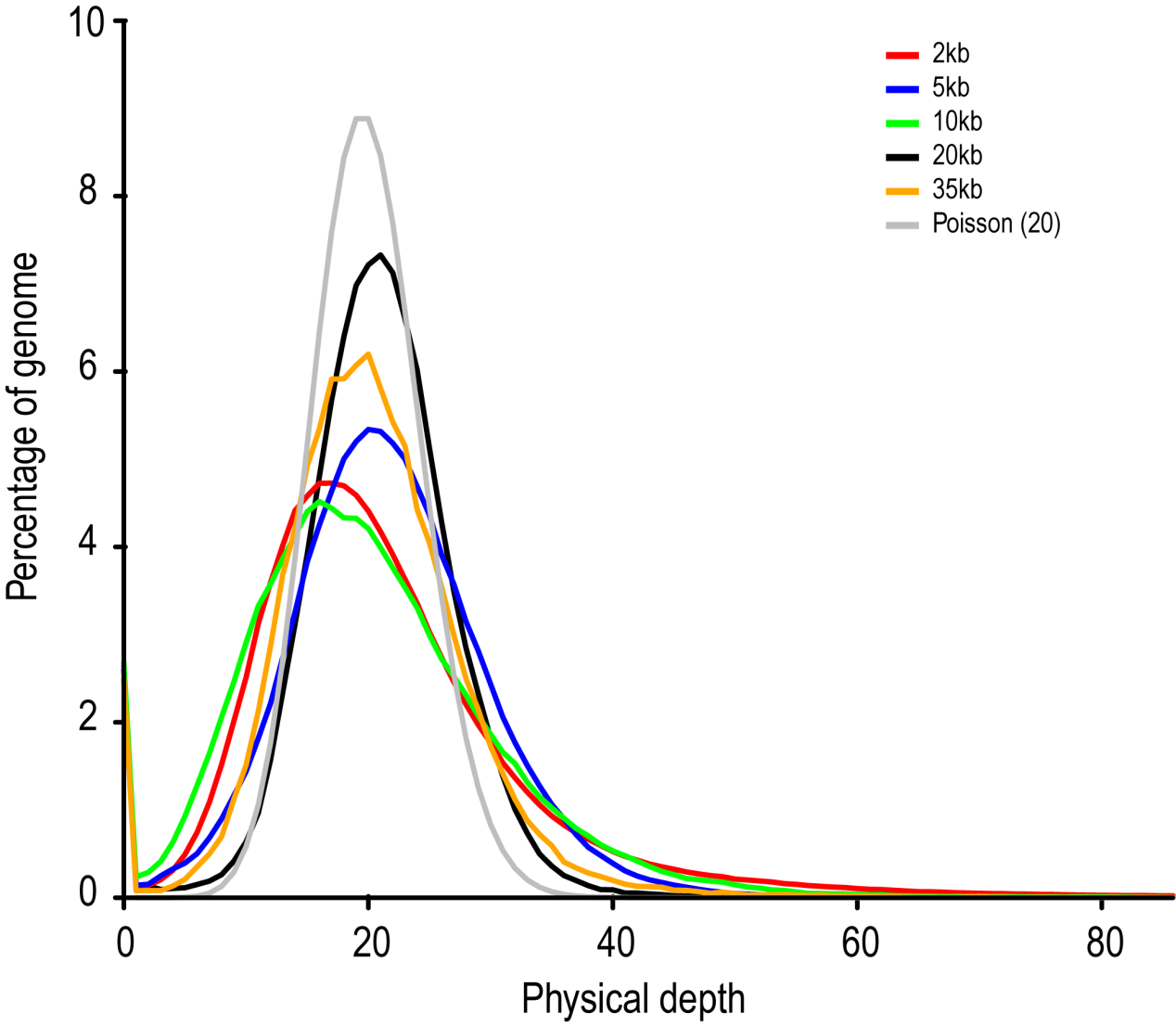


**Figure S2 physical coverage distribution of long-range PE libraries data on human genome.** A subset data of properly mapped unique read pairs that represent ~20-fold physical coverage on the human genome for each 2-kb (red), 5-kb (blue), 10-kb (green), 20-kb (black) and 35-kb libraries (orange) was sampled (base on the total data of 35-kb library) the analysis.Poisson (no bias) distributions (gray) with λ = 20 is also shown. Poisson distribution is the expected if there were absolutely no bias.
