# Supplementary material for: Paired-End Sequencing of Long-Range DNA Fragments for De Novo Assembly of Large, Complex Mammalian Genomes by Direct Intra-Molecule Ligation
Source: PLoS One. 2012 Sep 27;7(9):e46211. doi: 10.1371/journal.pone.0046211 (PMC3459883; doi:10.1371/journal.pone.0046211)
Supplement: Figure S3 — Alignments between the YH scaffolds and the reference human genome (Chr1–7, 9–22, X and Y). (DOC) [file pone.0046211.s003.doc]

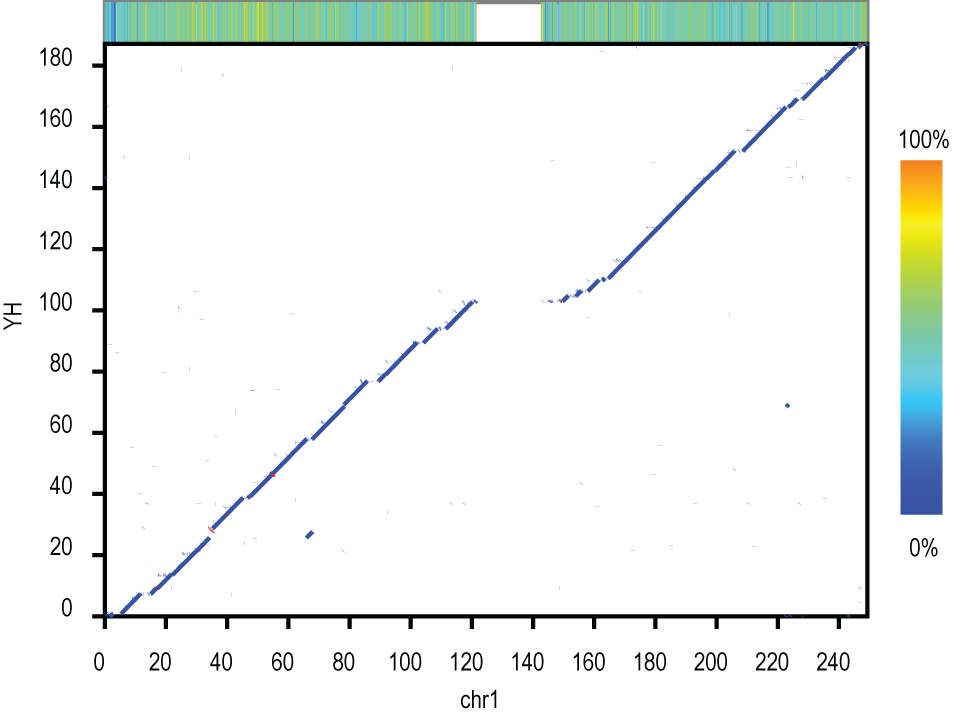

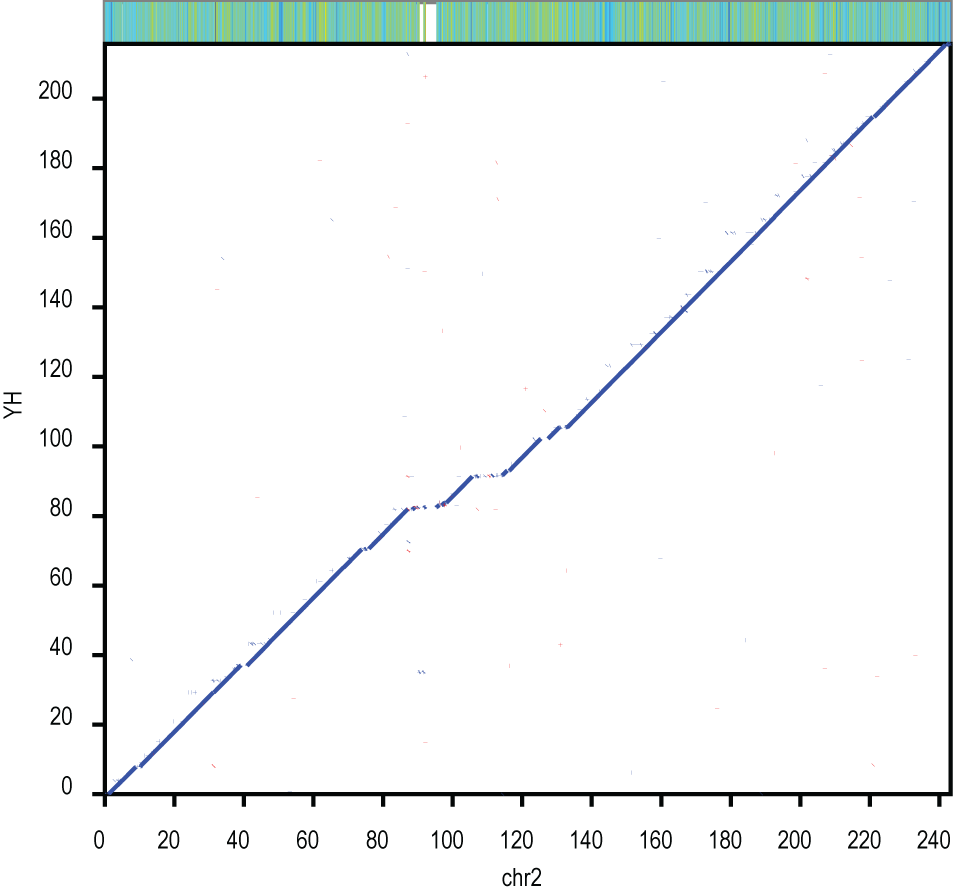

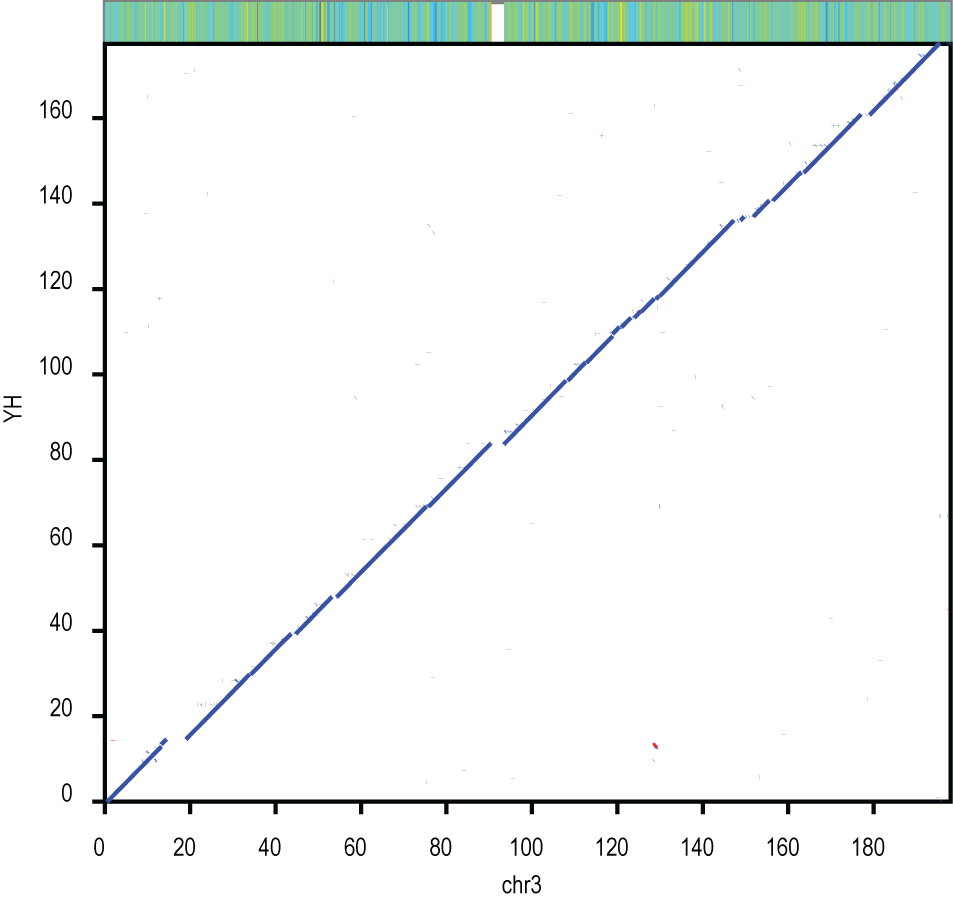

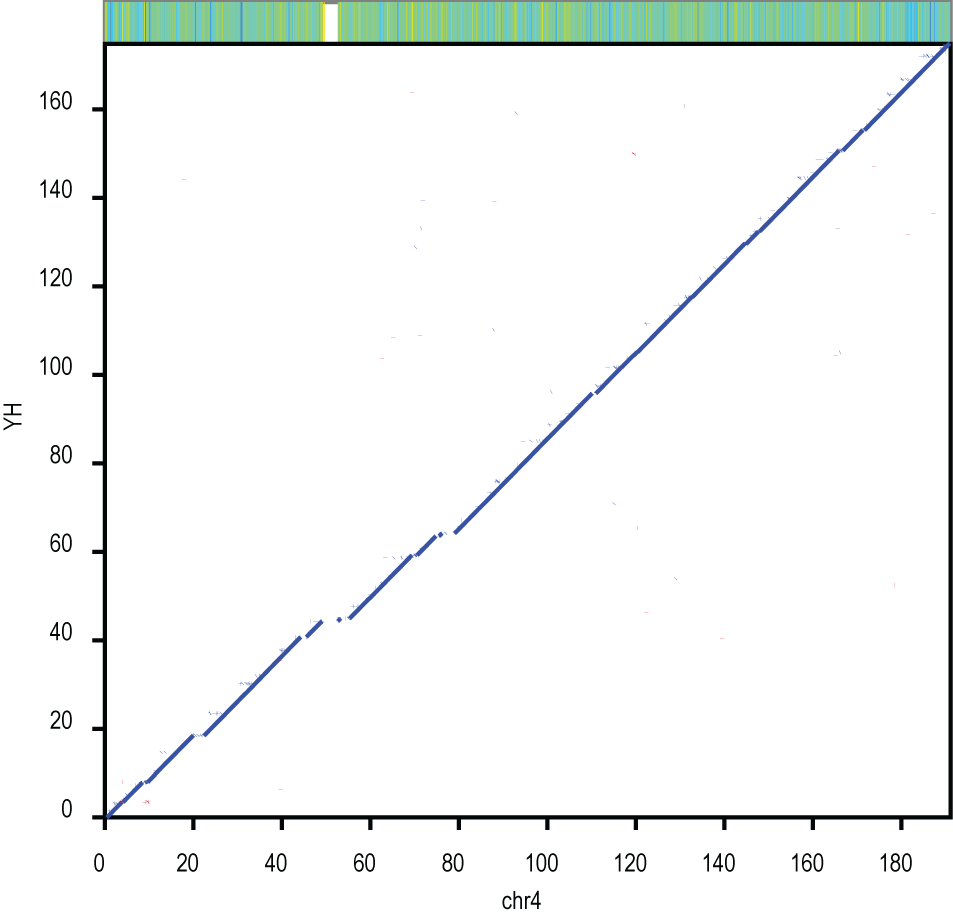

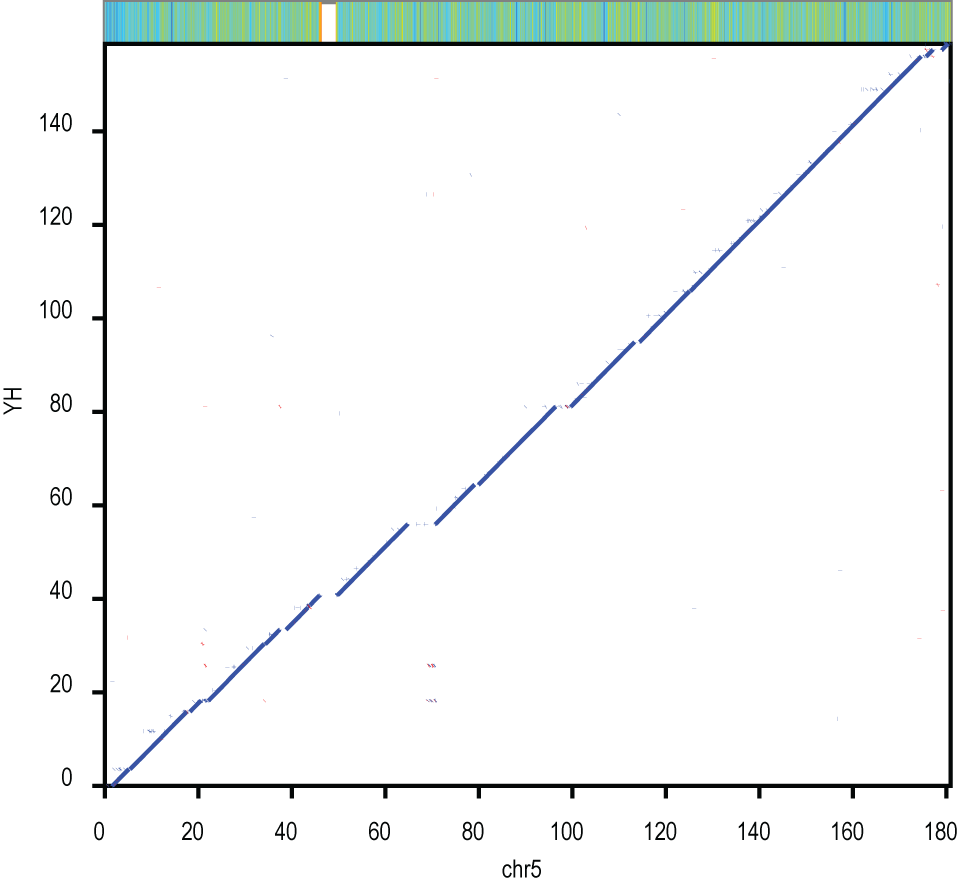

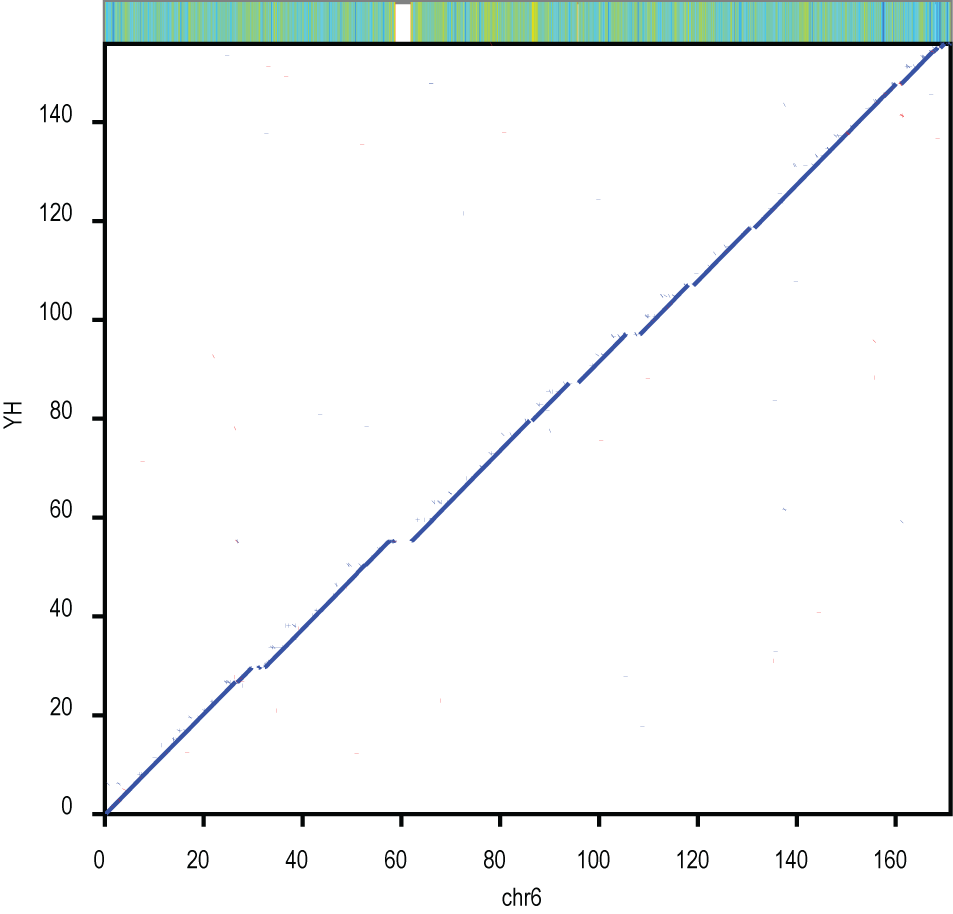

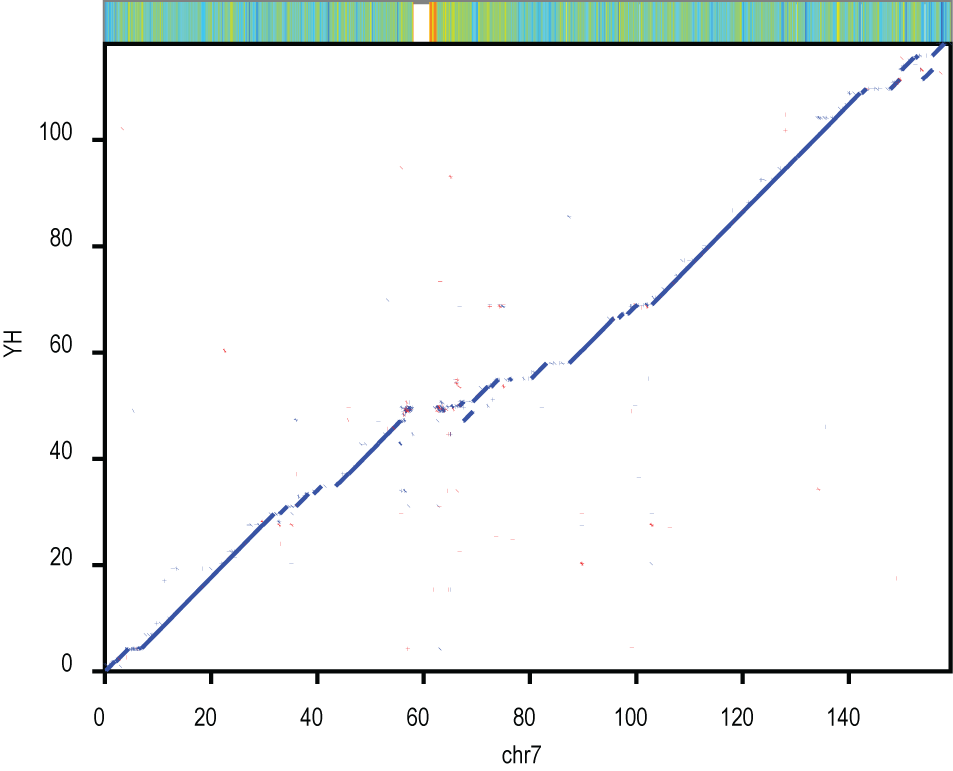

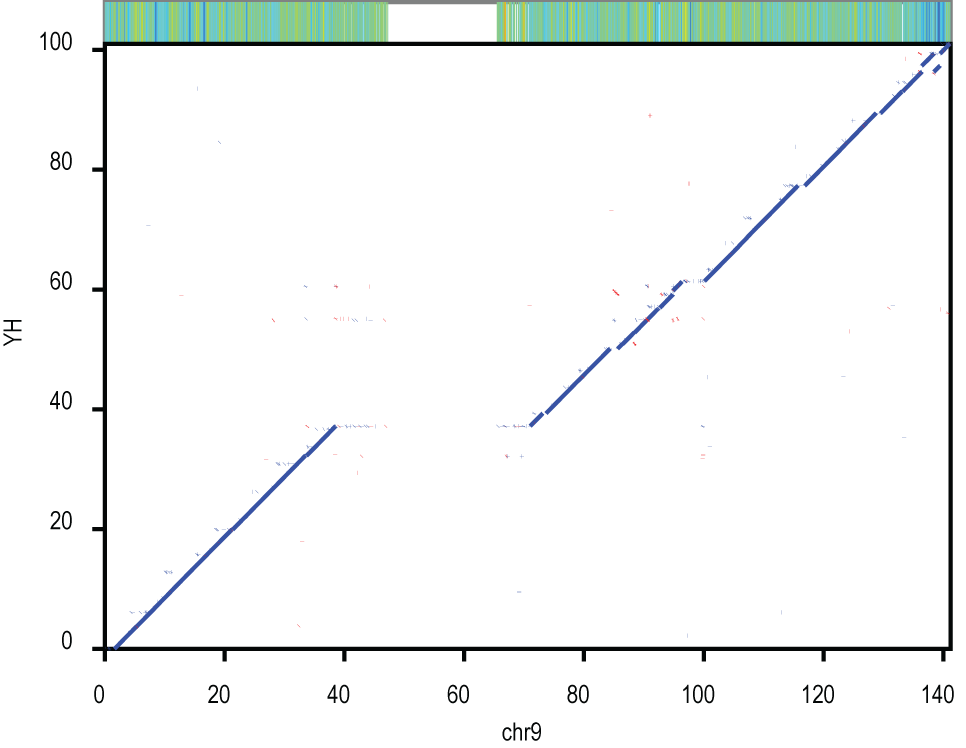

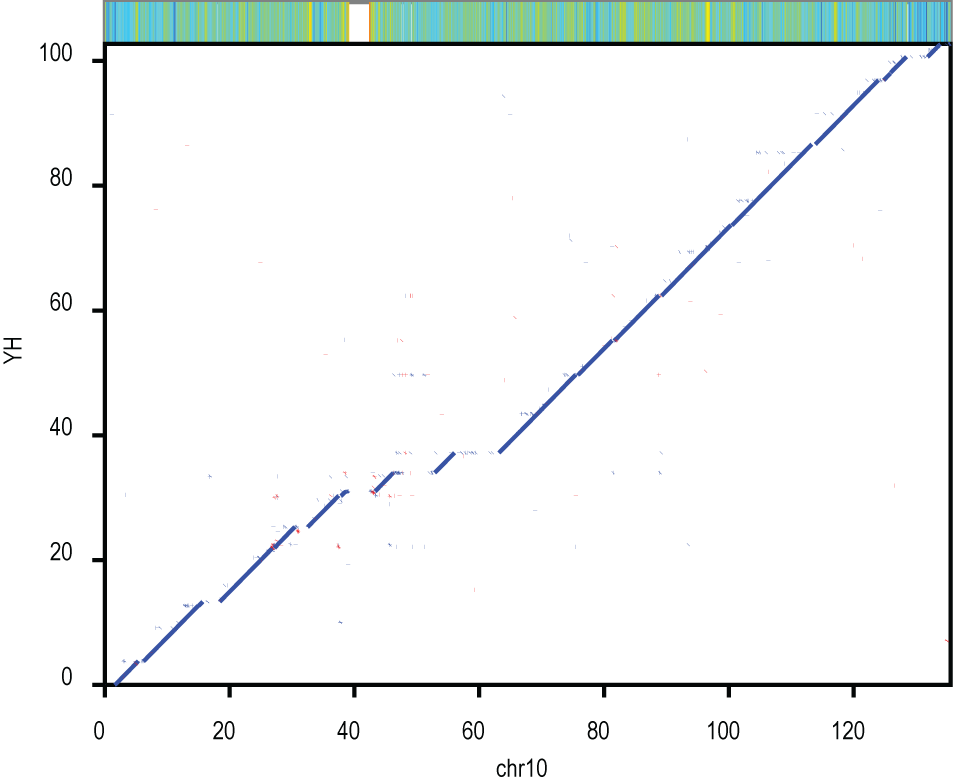

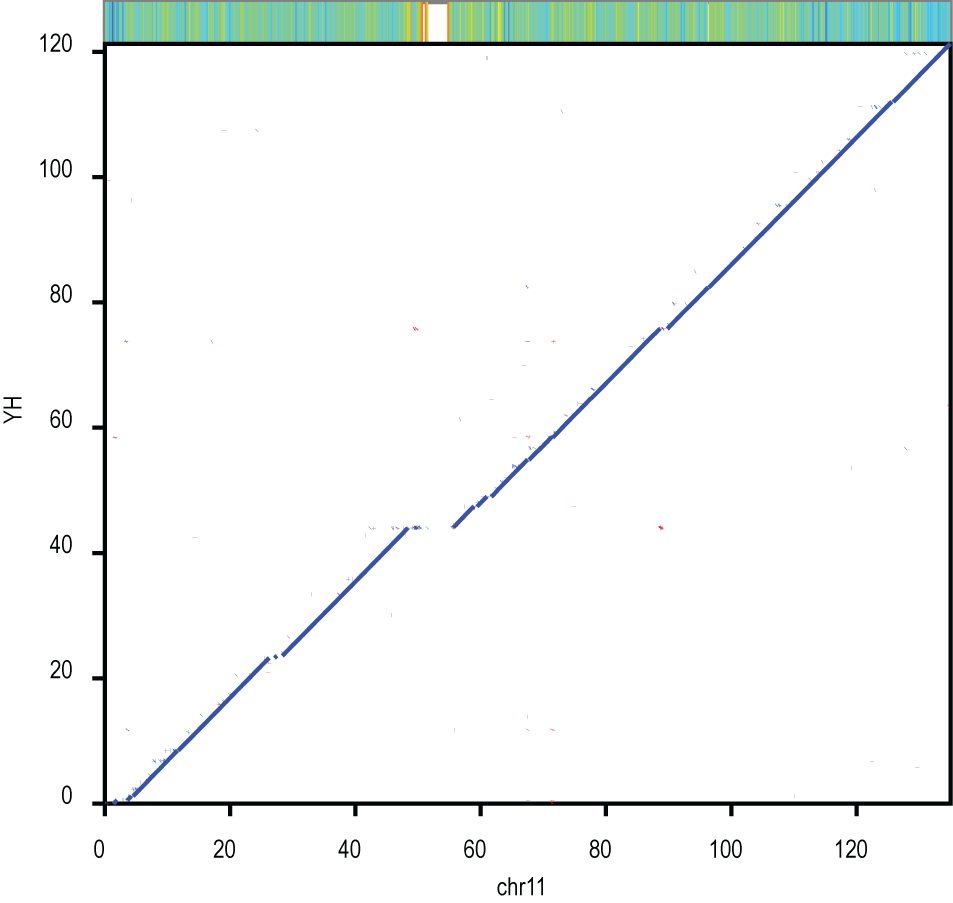

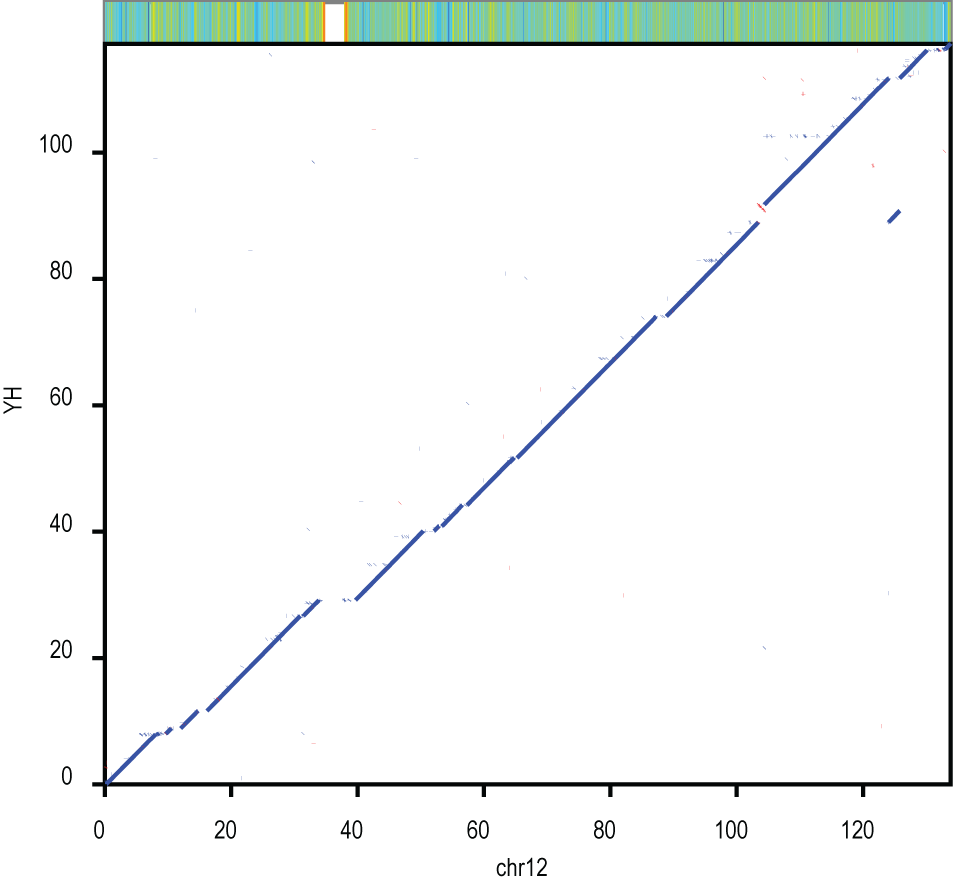

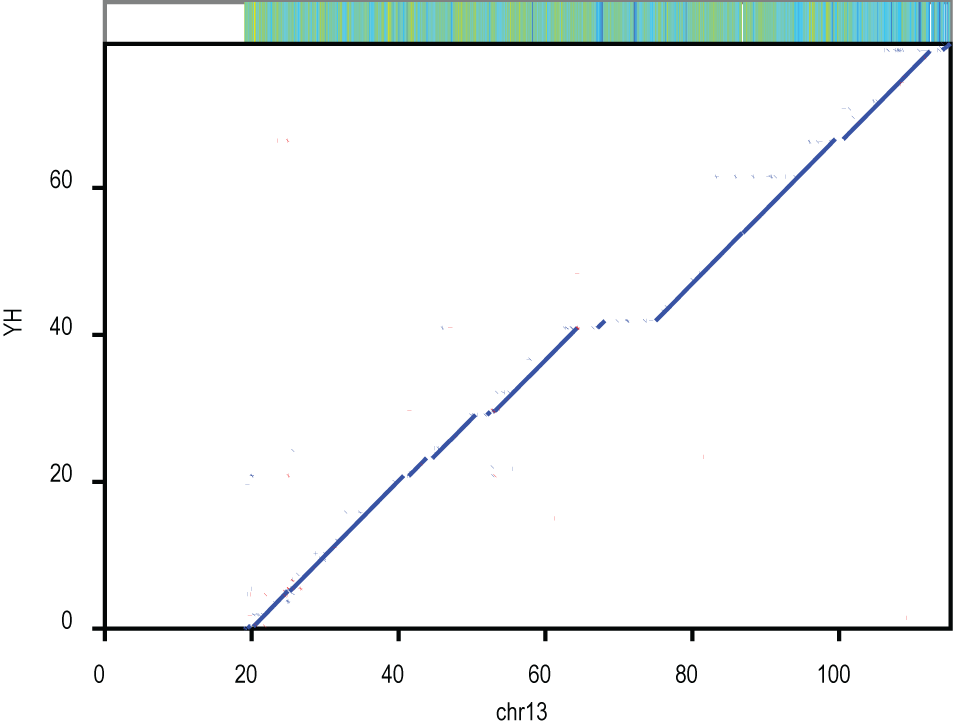

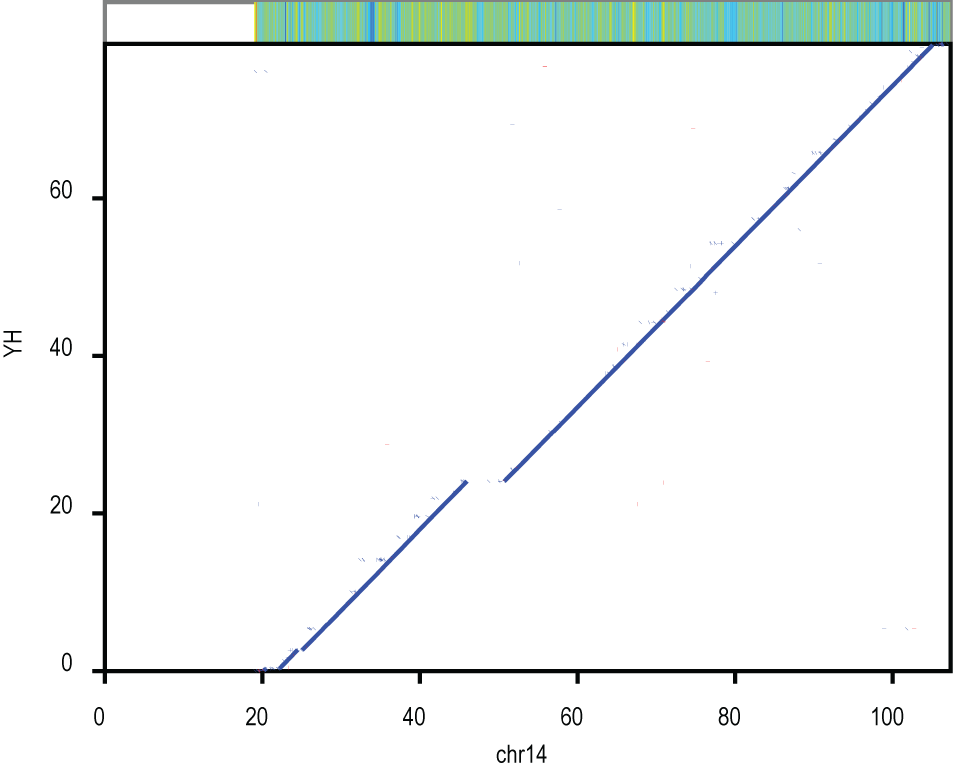

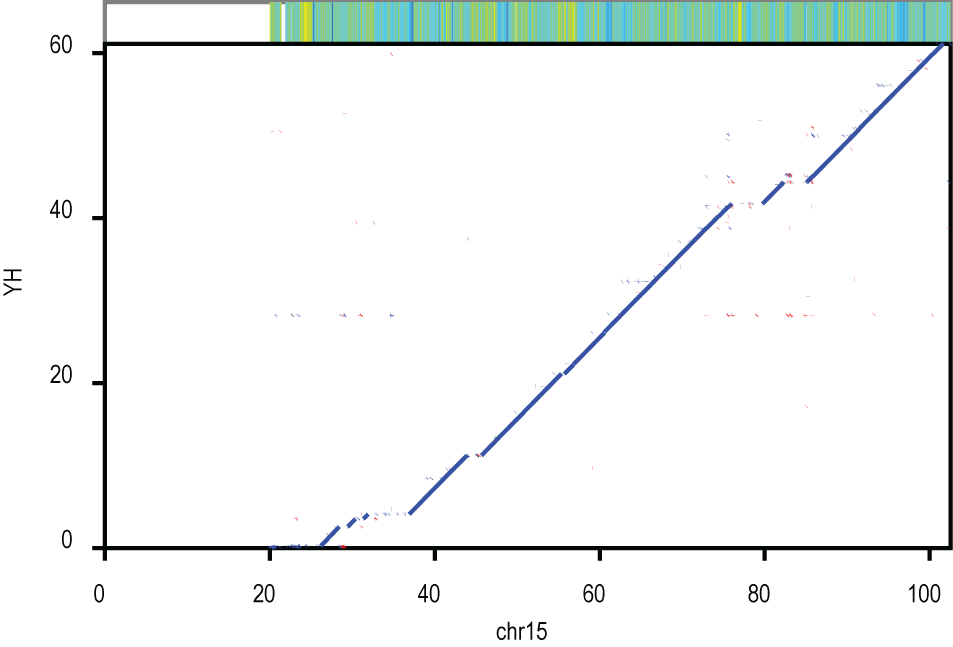

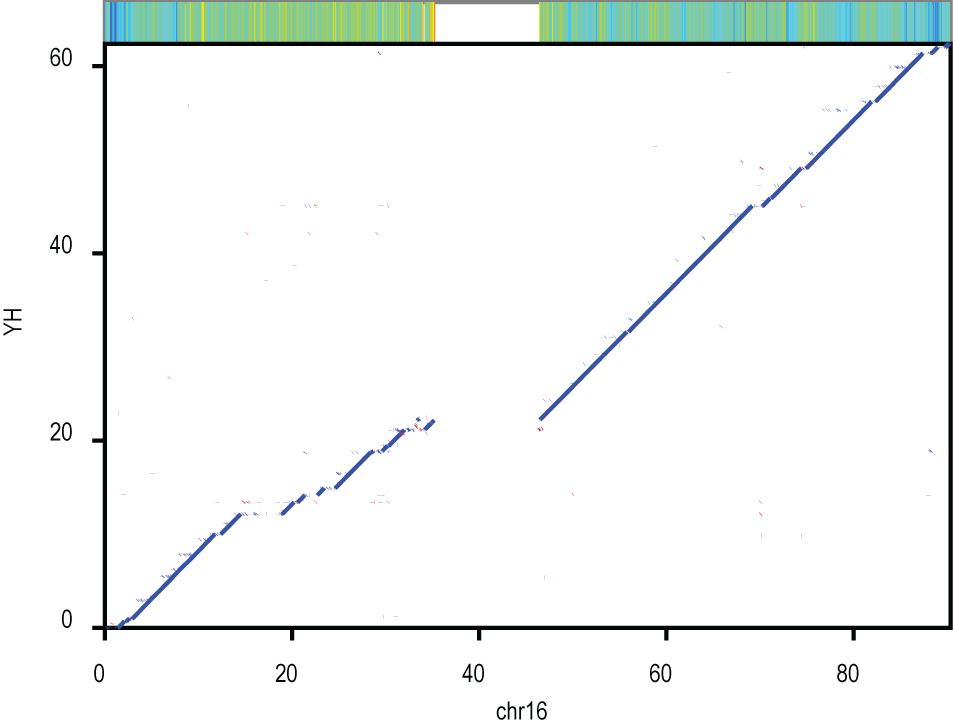

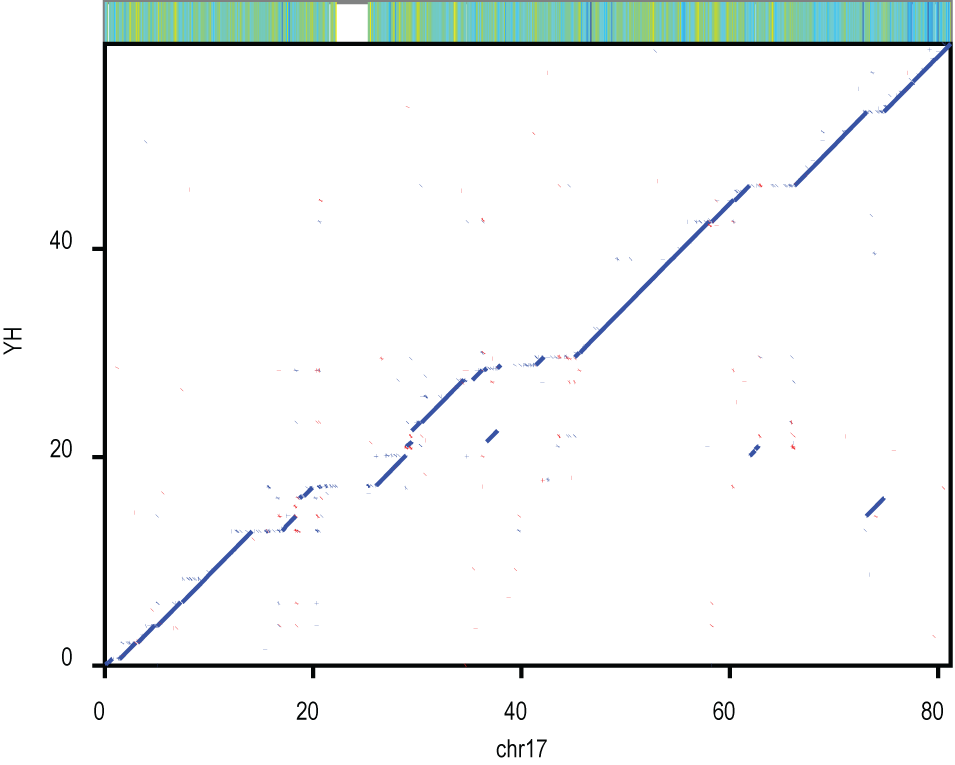

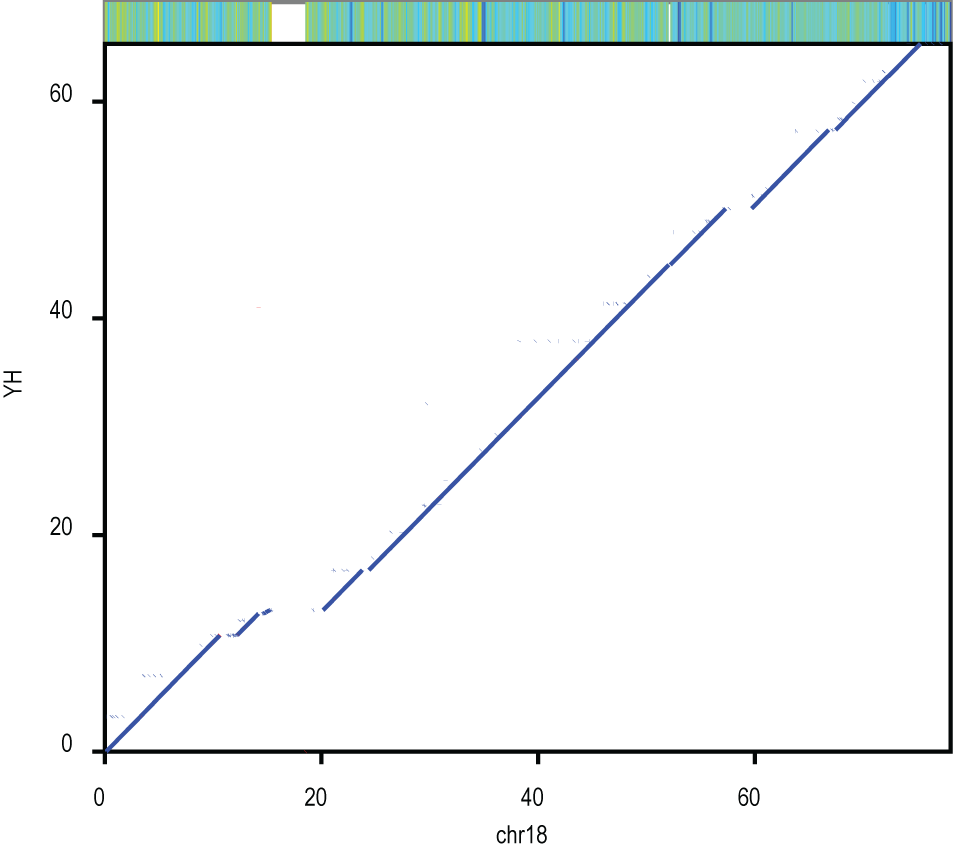

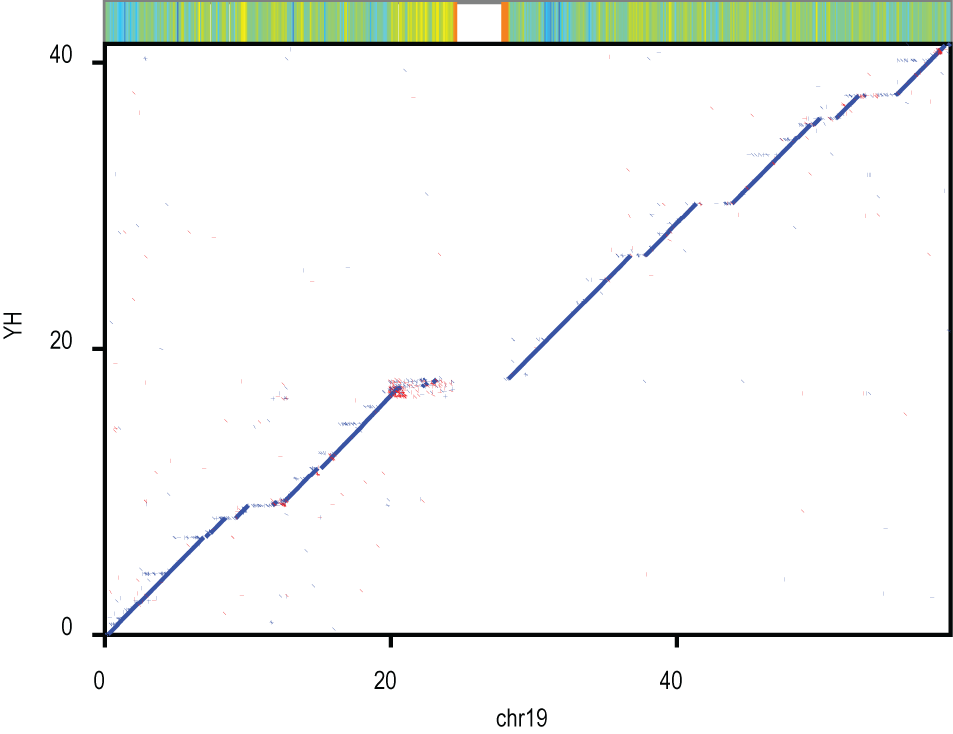

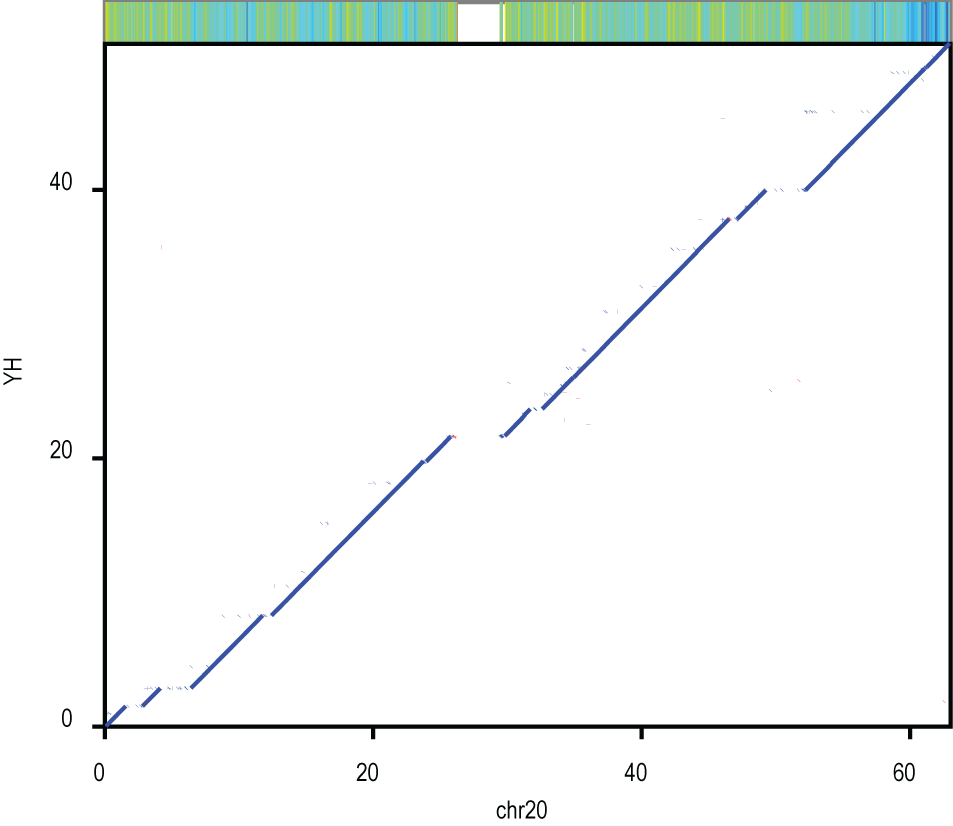

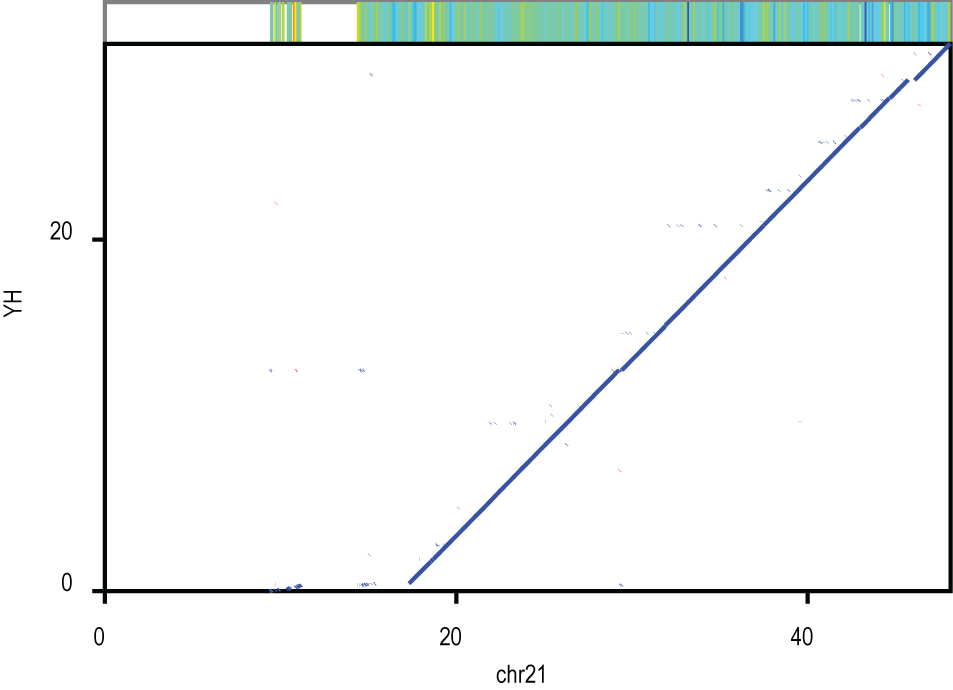

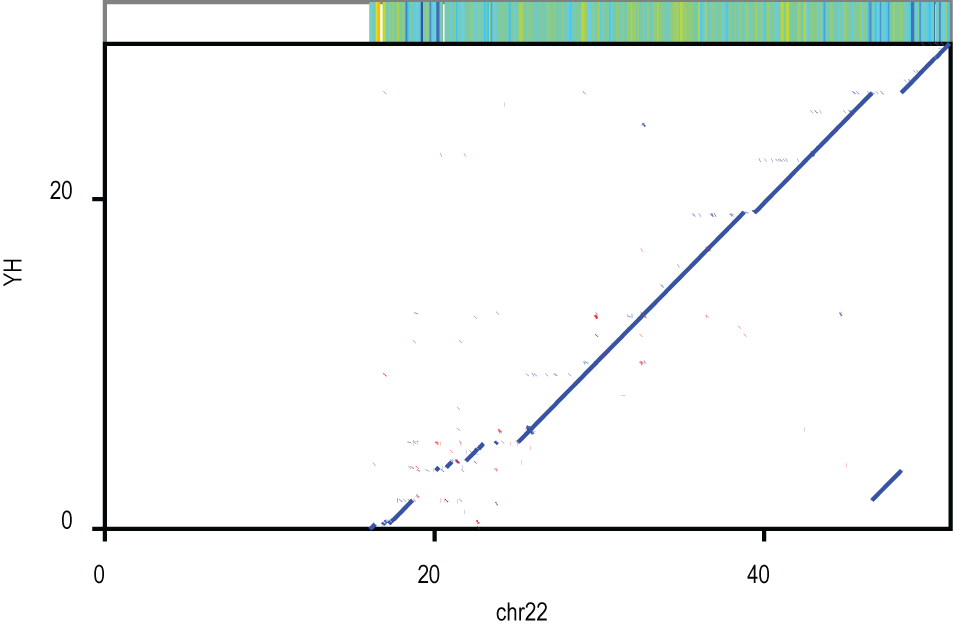

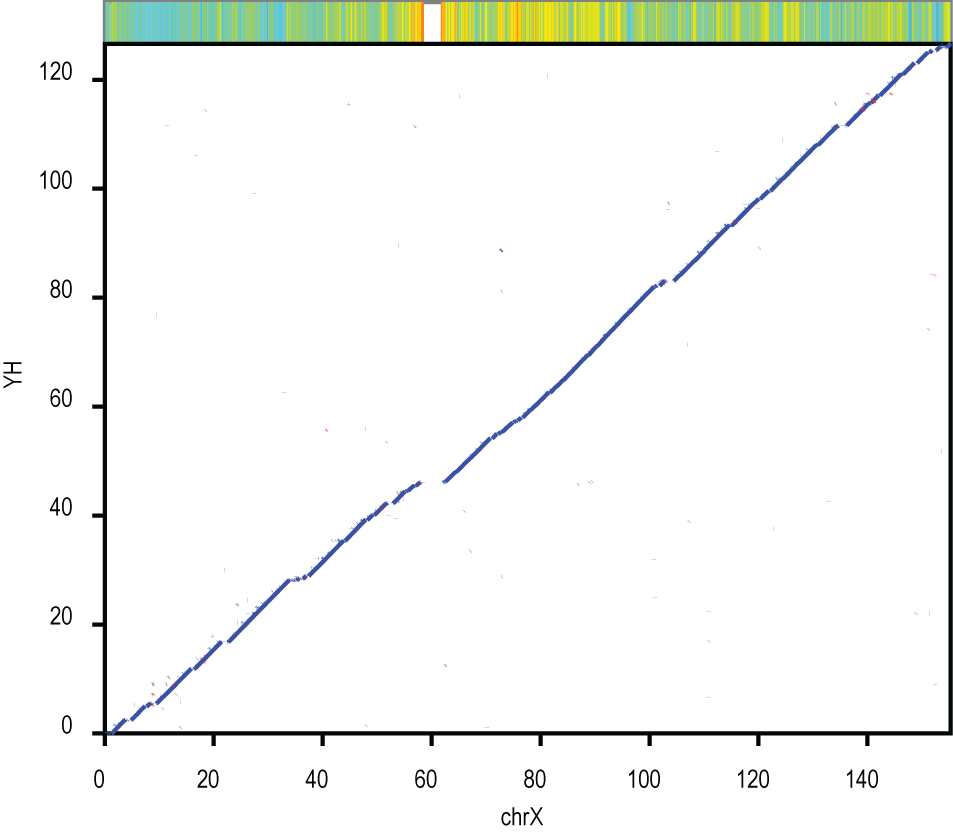

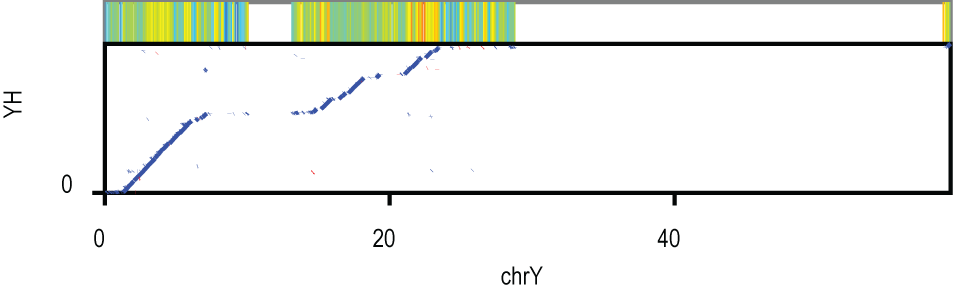


**Figure S3 Alignments between the YH scaffolds and the reference human genome (Chr1-7, 9-22, X and Y).** Local repeat level in each chromosome of NCBI build 37 (calculated in 1kb-window) was showed in color along the sequence at the top-up bar. The gaps in the reference genome were showed as white blocks.
