# Supplementary material for: Paired-End Sequencing of Long-Range DNA Fragments for De Novo Assembly of Large, Complex Mammalian Genomes by Direct Intra-Molecule Ligation
Source: PLoS One. 2012 Sep 27;7(9):e46211. doi: 10.1371/journal.pone.0046211 (PMC3459883; doi:10.1371/journal.pone.0046211)
Supplement: Figure S4 — PCR experimental validation of a newly identified ∼7-kb insertion in the YH genome chr14. (DOCX) [file pone.0046211.s004.docx]

**
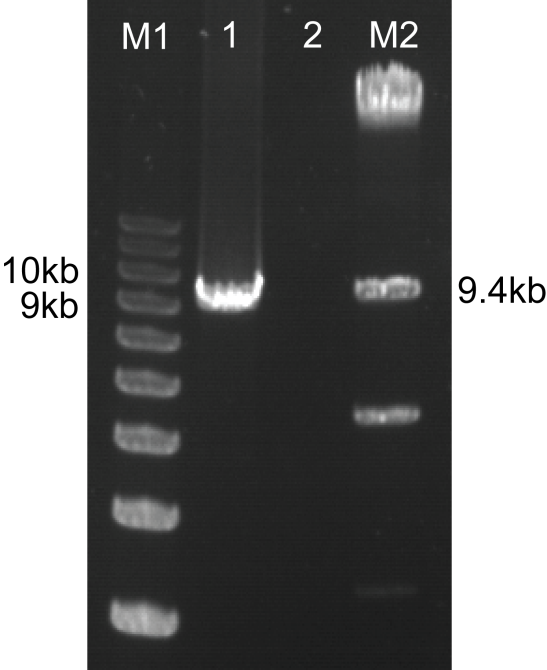
**

**Figure S4 PCR experimental validation of a newly identified ~7 kb insertion in the YH genome chr14.** A long-range PCR primer-pair was designed to cover a 2kb region in chromosome 14([chr14:50,226,449 to 50,228,422](http://genome.ucsc.edu/cgi-bin/hgTracks?hgsid=233561155&db=hg19&position=chr14:50226449-50228422&hgPcrResult=pack)) of the human reference genome (NCBI build 37) that covers the ~7kb insertion predicted from the long-range PE reads. The amplified PCR product size from the YH cell line DNA (~9kb) validated the 7kb insertion size (9kb minus 2kb) in (lane1). Lane 2, the negative control; lane M1 and M2, DNA markers.
